# Skewed X-Chromosome Inactivation and Compensatory Upregulation of Escape Genes Precludes Major Clinical Symptoms in a Female With a Large Xq Deletion

**DOI:** 10.3389/fgene.2020.00101

**Published:** 2020-03-04

**Authors:** Cíntia B. Santos-Rebouças, Raquel Boy, Evelyn Q. Vianna, Andressa P. Gonçalves, Rafael M. Piergiorge, Bianca B. Abdala, Jussara M. dos Santos, Veluma Calassara, Filipe B. Machado, Enrique Medina-Acosta, Márcia M. G. Pimentel

**Affiliations:** ^1^ Department of Genetics, Institute of Biology Roberto Alcantara Gomes, State University of Rio de Janeiro, Rio de Janeiro, Brazil; ^2^ Pedro Ernesto University Hospital, State University of Rio de Janeiro, Rio de Janeiro, Brazil; ^3^ Department of Biological Sciences, Minas Gerais State University, Ubá, Brazil; ^4^ Laboratory of Biotechnology, State University of Northern Rio de Janeiro Darcy Ribeiro, Rio de Janeiro, Brazil

**Keywords:** AR, escape genes, transcriptome-wide analysis, X-chromosome deletion, X-chromosome inactivation, RP2, transcriptome-wide analysis

## Abstract

In mammalian females, X-chromosome inactivation (XCI) acts as a dosage compensation mechanism that equalizes X-linked genes expression between homo- and heterogametic sexes. However, approximately 12–23% of X-linked genes escape from XCI, being bi-allelic expressed. Herein, we report on genetic and functional data from an asymptomatic female of a Fragile X syndrome family, who harbors a large deletion on the X-chromosome. Array-CGH uncovered that the *de novo*, terminal, paternally originated 32 Mb deletion on Xq25-q28 spans 598 RefSeq genes, including escape and variable escape genes. Androgen receptor (*AR*) and retinitis pigmentosa 2 (*RP2*) methylation assays showed extreme skewed XCI ratios from both peripheral blood and buccal mucosa, silencing the abnormal X-chromosome. Surprisingly, transcriptome-wide analysis revealed that escape and variable escape genes spanning the deletion are mostly upregulated on the active X-chromosome, precluding major clinical/cognitive phenotypes in the female. Metaphase high count, hemizygosity concordance for microsatellite markers, and monoallelic expression of genes within the deletion suggest the absence of mosaicism in both blood and buccal mucosa. Taken together, our data suggest that an additional protective gene-by-gene mechanism occurs at the transcriptional level in the active X-chromosome to counterbalance detrimental phenotype effects of large Xq deletions.

## Introduction

For dosage compensation of X-linked genes expression between hetero- (XY males) and homogametic (XX females) sexes, mammalian females have evolved a complex epigenetic mechanism to transcriptionally silence all but one X-chromosome per diploid set, called X-chromosome inactivation (XCI). In this process, which occurs in early embryogenesis, parental X-chromosomes have the same probability for random inactivation, giving rise to an overall 1:1 ratio of cells that express either the paternal or the maternal X-chromosome. Once XCI has occurred, the inactive X-chromosome (Xi) is stably transmitted through subsequent mitosis. Nonetheless, non-random or skewing of XCI can arise by chance or due either to primary nonrandom choice or to secondary stochastic or genetic processes (Fieremans et al., 2016). In primary skewing, variants in genes participating from the XCI process itself (i.e., *XIST*) preclude the cell from silencing the X-chromosome carrying the mutation before the XCI starts. Alternatively, secondary skewing generally takes place in post-inactivation cell selection, acting for or against cells carrying the active X-chromosome (Xa) or the Xi (Morey and Avner, 2011). So, secondary XCI skewing often occurs in females with a structurally abnormal X-chromosome, such as large deletions, duplications, and unbalanced X/autosome translocations, in a manner that preserves the normal X-chromosome and autosomal dosage (Schmidt et al., 1991). Conversely, in balanced X/autosome rearrangements, the normal X-chromosome is usually inactive, in order to keep functional euploidy (McMahon and Monk, 1983).

Cumulative evidence also estimates that 12–23% of X-linked genes in humans escape from XCI, being expressed from both the Xa and Xi (Carrel and Willard, 2005; Balaton et al., 2015; Tukiainen et al., 2017). XCI escape genes are distributed in clusters, mainly located on the short arm of the X-chromosome, possibly as a reflection of their distance from the XCI center (Xic) (Disteche, 1999; Tsuchiya et al., 2004; Carrel and Willard, 2005). Besides, one intrigant cluster of Xi-expressed genes maps in a gene-rich region at Xq28 (Carrel and Willard, 2005).

It is noteworthy, however, that genes located on the human X-chromosome seem to be expressed in few tissues or are specific for a subset of tissues, e.g., brain (Hurst et al., 2015). Furthermore, there is an excess of XCI escape genes involved in neurocognitive function (Zhang et al., 2013), which could explain some of the somatic abnormalities seen in females and males with sex chromosome aneuploidies like Turner or Klinefelter syndromes, even in the presence of only one Xa. Moreover, intellectual disability (ID) is a common phenotypic component among females harboring mutations on escape genes and XCI skewing (Fieremans et al., 2015; Snijders Blok et al., 2015; Fieremans et al., 2016; Reijnders et al., 2016).

Herein, we report a female with a *de novo* heterozygous deletion at Xq25-q28 associated with an extreme XCI skewing pattern against the deleted X-chromosome. The patient was evaluated due to the presence of Fragile X syndrome (FXS; MIM# 300624) in her nephew. Surprisingly, transcriptome analysis revealed an upregulation compensatory mechanism of X-linked genes within the deleted region that escape or variable escape XCI, including ID genes, preventing her from having ID and/or other major clinical features, but premature ovarian failure (POF). Altogether, our data suggest that, at least for some XCI escape genes, structural hemizygosis caused by large X-chromosome deletions may be transcriptionally counterbalanced, avoiding functional haploinsufficiency.

## Materials and Methods

### Study Participants

**Figure 1:**
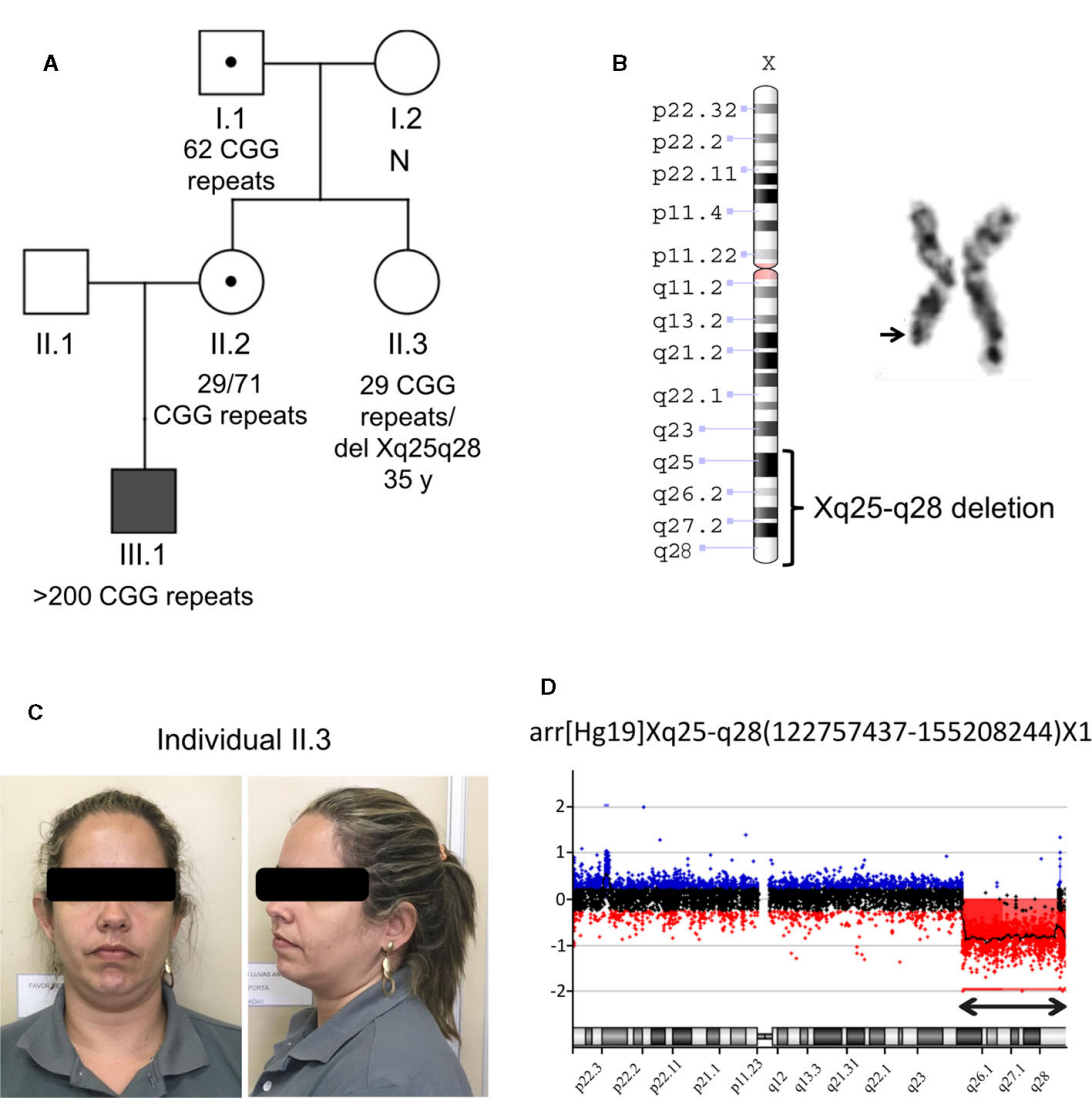
Molecular and cytogenetics analysis in the studied family. **(A)** Family pedigree showing the segregation of the *FMR1* CGG repeat expansion that was ascertained through the proband with ID (individual III.1 indicated by a solid square). Open squares represent unaffected males and open circles represent unaffected females. Circle or square with a black dot represents an unaffected carrier female or male, respectively. “N” indicates no *FMR1* expansion. A heterozygous Xq25-q28 deletion is present in the individual II.3; **(B)** Partial G banded karyotype from the individual II.3 and ideogram of the Xq deleted region. **(C)** Pictures of the individual II.3 that harbors the Xq25-q28 deletion; **(D)** X-chromosome array-CGH analysis plot from the individual II.3. Cy3-labeled DNA of the individual II.3 was co-hybridized with Cy5-labeled DNA from a control onto the array. The double arrow points to the deletion of subsequent probes. Note that the deletion is seen as an increased Cy5/Cy3 ratio.

The research protocols adhered to the ethical principles for medical research involving human subjects and received approval from the Institutional Ethics Committee. The index family was referred to the Human Genetics Laboratory at the State University of Rio de Janeiro (Rio de Janeiro, Brazil) in 2016, because of an idiopathic history of ID and autism in the propositus, compatible with FXS. The three-generation family comprised five members available for testing (individuals I.1, I.2, II.2, II.3, III.1), including the asymptomatic aunt of the proband (individual II.3), who was tested as part of a routine genetic counseling procedure for FXS (**Figure 1A**).

### FMR1 Analysis

For molecular analysis, genomic DNA was isolated from peripheral blood samples from available family members. High-resolution methylation PCR (mPCR) on the proband (III.1) was performed using AmplideX *FMR1* mPCR kit (Asuragen Inc., Austin, TX, USA). For *FMR1* expansion segregation analysis, the mother (II.2), the maternal aunt (II.3) and the maternal grandparents (I.1 and I.2) were also evaluated by AmplideX *FMR1* mPCR kit (Asuragen Inc., Austin, TX, USA) (Gonçalves et al., 2016).

### Karyotype and Array-Comparative Genomic Hybridization (array-CGH)

In parallel to FXS interrogation, cytogenetic evaluation was performed on cultured peripheral blood lymphocytes from the proband, by standard methods to exclude chromosome aberrations linked to ID. As the proband's aunt (II.3) expressed the intention of becoming pregnant, standard karyotype analysis was also performed in her peripheral blood cells.

With the purpose of delineating an Xq deletion detected in individual II.3 karyotype (**Figures 1B, C**), array-CGH was conducted in gDNA extracted from her peripheral blood using a 180 K whole-genome platform (Agilent Technologies, Santa Clara, CA, USA). Samples were labeled with Cy3- and Cy5-deoxycytidine triphosphates by random priming. Purification, hybridization, washing, image scanning, and data analysis were carried out as previously reported (Santos-Rebouças et al., 2015).

### Microsatellite Genotyping and X-Chromosome Inactivation Assay

To assess the parental origin of the Xq deletion, six polymorphic microsatellite repeat markers along the X-chromosome were interrogated in all family members available by quantitative fluorescence PCR using fluorochrome-labeled primers and separating the amplimers by high-resolution capillary electrophoresis, as previously described (Ogilvie et al., 2005). Both blood and buccal mucosa DNA samples were genotyped. Three heterozygous microsatellites within the Xq deletion were informative to confirm the parental origin of the deletion.

Besides, the extent of XCI was estimated by determining the Xa/Xi ratios in DNA from blood, and buccal mucosa of individual II.3 using the methylation-sensitive restriction enzyme indirect *AR*/*RP2* biplex assay previously reported (Machado et al., 2014). Allele profiles and areas under the curve for each allele were determined on an ABI3130 Genetic Analyzer (Thermo Fisher Scientific Inc., MA, USA) and data were analyzed by GeneScan Analysis 3.7 and Genotyper 3.7 software (Thermo Fisher Scientific Inc.). Fluorescent peak areas representing true alleles were normalized for the existence of stutter products, and the XCI ratios were estimated as previously described (Busque et al., 2009; Machado et al., 2014).

### RNA-Seq

Blood RNA samples from individual II.3 and an age and sex-matched control were subsequently analyzed by RNA-Seq in Illumina platform (Genone Biotechnologies, Rio de Janeiro, Brazil). Total RNA was purified using poly-T oligo-attached magnetic beads with rRNA removal. The resulting directional RNA-Seq NEB libraries were sequenced in paired-end format. Image analysis and per-cycle base calling were performed with Illumina Real-Time Analysis software (RTA1.9) (Illumina). Conversion to FastQ read format was obtained by CASAVA-1.8 (Illumina) and sequenced reads were quality-checked with FastQC (Andrews, 2010). Sequence adaptors were removed with cutadapt v1.2.1 (Martin, 2011), and reads were aligned to the human reference genome (GRCh37/hg19) with STAR (Dobin et al., 2013). BAM files were visualized by using the Integrative Genomics Viewer (IGV) (Robinson et al., 2011).

For single nucleotide polymorphisms (SNPs) and insertions/deletions (indels) analyses, Samtools and Picard were used to sort the reads according to the genome coordinates, followed by screening out repeated reads. Finally, GATK3 (McKenna et al., 2010) was used to carry out SNP and indel calling. ANNOVAR (Wang et al., 2010) was applied for annotation and variants were reported according to the Human Genome Variation Society (HGVS) guidelines for cDNA sequence variants (GRCh37/hg19).

For differential expression analysis, HTSeq v0.6.1 (Anders et al., 2015) was used to count the read numbers mapped for each gene and Fragments Per Kilobase of transcript sequence per Millions base pairs sequenced (FPKM) were used to estimate gene expression levels, taking into consideration the effects of both sequencing depth and gene length on the counting of fragments (Mortazavi et al., 2008). Subsequently, read counts were adjusted by TMM, then differential expression analysis was performed by using the EdgeR R package (Robinson et al., 2010). In the absence of biological replicates, adjusted p-value or q value < 0.005 and absolute fold change of 1 were set as the threshold for significant differential expression. The distribution of the differentially expressed genes was depicted using Volcano plots.

ClusterProfiler (Yu et al., 2012) and Enrichr software (Chen et al., 2013; Kuleshov et al., 2016) were used for enrichment analysis of the differential expressed genes, including Gene Ontology (GO) Consortium (2004) and Human Disease Ontology (DO) enrichement (Schriml et al., 2019), and adjusted p-values < 0.05 were indicative of significant enrichment.

TFCat (Fulton et al., 2009) was used for searching transcription factors (TF) for the differential expressed genes. Besides, identification of oncogenes and their annotation was done by searching the Catalogue Of Somatic Mutations In Cancer (COSMIC) database (Tate et al., 2019) using the differentially expressed genes.

Replicate multivariate analysis of transcript splicing (rMATS) (Shen et al., 2014) was used for detection of differential alternative splicing from RNA-Seq data, identifying skipped exon (SE), alternative 5' splice site (A5SS), alternative 3' splice site (A3SS), mutually exclusive exons (MXE), and retained intron (RI) events. The threshold of significant difference in alternative splicing analysis was set at FDR < 0.01.

To evaluate the expression of genes within the Xq deletion, we performed differential expression (DE) analysis using only genes that were expressed at a mean level above 10 counts per million (CPM) at least in one sample (individual II.3 or control). For comparing more accurately expression levels of blood-expressed genes within the deletion and minimize transcriptional variance between individuals, we searched for additional RNA-Seq experiments from healthy controls on Sequence Read Archive (SRA; https://www.ncbi.nlm.nih.gov/sra), using “control”, “blood”, “HiSeq 2500”, “RNA-Seq”, “paired”, as search terms for type of samples, tissue, instrument, assay-type, and library layout, respectively. Four female (SRR3745154, SRR3745158, SRR3745160, SRR3745166) and one male (SRR3745151) samples from adult individuals residing in the same geographic area of individual II.3 and control (Rio de Janeiro, Brazil) were selected [Bioproject: PRJNA327986; (de Araujo et al., 2016); personal communication]. Besides, we included two RNA-Seq samples from additional healthy males (SRR3389246, SRR3390437) described on the Bioproject PRJNA316578 (no associated publication). Two DE comparisons were performed: individual II.3 versus males (SRR3745151, SRR3389246, SRR3390437) (group 1) and individual II.3 versus females (our control, SRR3745154, SRR3745158, SRR3745160, SRR3745166) (group 2). Only genes within the deletion with a CPM over ten (individual II.3 or our control) were evaluated in these latter DE comparisons, using the same pipeline described above.

## Results

A paternal large deletion was identified in the terminal part of the long arm of the X-chromosome (Xq25-q28) in the aunt of the proband with Fragile X syndrome. POF is the unique apparent phenotype in this female. Using methylation assays in blood and buccal mucosa, we showed that extreme XCI skewing resulted in the silencing of the structurally abnormal X-chromosome. Besides, focusing on the genes located within the deletion, transcriptome analysis of blood samples from this female in comparison to matched controls revealed that genes annotated as escape or variable escape genes are upregulated, preventing major clinical phenotypes in this individual. The application of different assays described below excluded the possibility of mosaicism.

### FMR1 Analysis

mPCR in the family of the female harboring the Xq deletion confirmed a fully methylated expansion in the *FMR1* gene on her nephew (proband III.1; > 200 CGG repeats), compatible with the FXS phenotype. As expected, segregation analysis in the mother of the proband (individual II.2) showed a *FMR1* methylated premutation (normal allele: 29 CGGs; expanded allele: 71 CGGs), that was inherited from her father, individual I.I, who has a smaller unmethylated premutated allele (62 CGGs). Subsequent *FMR1* gene CGG repeat number evaluation in the proband's aunt (individual II.3), the female explored in details in this study, revealed the presence of only one unmethylated allele with 29 repeats, suggesting homozygosity for the *FMR1* CGG triplets (**Figure 1A**; **Supplementary Figure S1**).

### Cytogenetic and Array-CGH Findings

Cytogenetic evaluation in the aunt (individual II.3) for genetic counseling purposes by standard karyotype revealed a large terminal deletion on the long arm of X-chromosome (Xq25-q28) (**Figures 1B, C**). One hundred metaphases were analyzed, which excludes the hypothesis of mosaicism. Oligo array-CGH revealed a hemyzygous deletion of at least 32,450,808 bp (chrX:122,757,437-155,208,244; hg19), comprising 598 NCBI RefSeq curated genes, pseudogenes and microRNAs (**Figure 1D**). According to the array-CGH, the proximal breakpoint of the terminal deletion is within the *THOC2* gene, and no other potential pathogenic CNV was found. The rearrangement reported in this study has been submitted to ClinVar (https://www.ncbi.nlm.nih.gov/clinvar/) with accession number SCV000897650.

### Individual II.3 Phenotype

Individual II.3 was first evaluated at 34 years old. She is the second daughter of a nonconsanguineous couple and her hallmark developmental milestones did not point to delayed cognitive functioning or unexpected adaptive skills abnormalities. She holds a Bachelor's degree in Biological Science, and she currently works in the administrative sector of a private enterprise, with no apparent mild cognitive impairment or other major clinical condition (**Figure 1C**). Cranial Magnetic Resonance imaging performed in 2016 presented normal results. After the detection of the Xq25q28 deletion, she searched for an IVF for reproductive assistance. During the process, she began having irregular menses, and routine biochemical tests revealed abnormal anti-Mullerian hormone (<0.001 ng/ml), follicle-stimulating hormone (73.4 mUI/ml), and luteinizing hormone (33.6 mUI/ml) levels, compatible with early menopause. Videohysteroscopy showed endocervical polyps, normal uterine cavity, and atrophic endometrial. Her family history is negative for either infertility or premature ovarian insufficiency/failure (POI/POF). Currently, she is considering *in vitro* fertilization with egg donation.

### Parental Origin of the Xq Deletion and XCI Patterns

Parental origin of the abnormal X-chromosome in the family, assessed with linkage analysis with highly polymorphic microsatellite *loci* along this chromosome, showed that the Xq25-q28 deletion in individual II.3 occurred in the germline of her father (I.1). Both blood and buccal mucosa DNA samples showed complete hemizygosity for the DNA markers within the deletion (**Supplementary Table S1**).

Methylation-sensitive restriction enzyme typing with the *AR*/*RP2* biplex assay proxy of XCI revealed extreme skewing (>90%) for both *AR* and *RP2* gene markers in blood and buccal mucosa (**Supplementary Figure S2** and **Table S2**). The preferential XCI turned off the abnormal X-chromosome (236 bp allele for *AR* and 374 bp allele for *RP2*), inherited from his father (individual I.1).

### RNA-Seq

Blood RNA-Seq data quality summary is found on **Supplementary Table S3**. Reads across four highly polymorphic and high-quality SNPs within the deletion demonstrated monoallelic expression, suggesting no detectable mosaicism and confirming the near to complete XCI skewing observed in the blood sample of individual II.3 (**Supplementary Table S4**). No blood-expressed indels were found from the proximal array-CGH breakpoint until the end of X-chromosome (**Supplementary Table S5**).

Transcriptome-wide analysis in individual II.3 uncovered 1,026 differentially blood-expressed genes, as compared with the matched control sample (**Supplementary Figure S3**). From the 598 RefSeq genes mapping within the X-chromosome deletion, 241 genes were expressed on blood according to our RNA-Seq analysis. From these, 117 transcripts have more than 10 counts in at least in one of the samples (individual II.3 or control) (**Supplementary Table S6**). Only three genes within the deletion (*GPR112*, *SLC6A8*, and *FUNDC2*) showed statically significant adjusted p-values and | log2(FoldChange)|, for which *GPR112* (log2 fold change value = -7,72; q value = 0,0002), was underexpressed and *SLC6A8* (log2 fold change value = 1,88; q value = 0,001), and *FUNDC2* (log2 fold change value = 1,81; q value = 0,002), were overexpressed in individual II.3, in comparison to control. No differential expression was found for *THOC2* gene, located on the proximal breakpoint. Additional analysis of X-linked genes outside the deletion revealed 15 other genes differentially expressed (**Supplementary Table S7**).

Human Disease Ontology analysis showed that the differentially expressed genes are enriched in auditory system disease, proteinuria, primary ciliary dyskinesia, and idiopathic generalized epilepsy. None of these conditions are present in individual II.3 (**Supplementary Figure S4**). TFCat and COSMIC databases did not disclose any oncogene or transcription factor associated with the differentially expressed genes mapping within the Xq25-q28 deletion.

Global GO enrichment analysis revealed significant values for the three classes. The terms with the best scores (adjusted p-value < 0.01 and at least ten genes) for each category scored by p-value were represented in **Supplementary Figure S5**. Enriched Biological Processes were mainly related to homophilic cell adhesion *via* plasma membrane adhesion molecules, cell-cell adhesion *via* plasma-membrane adhesion molecules, membrane depolarization during action potential, synapse organization, extracellular matrix organization, extracellular structure organization, action potential, multicellular organismal signaling, regulation of membrane potential, and sensory perception of sound. Molecular Functions enriched terms encompassed motor activity, extracellular matrix structural constituent, actin binding, transmembrane receptor protein tyrosine kinase activity, calmodulin binding, dynein light chain binding, transmembrane receptor protein kinase activity, ATP-dependent microtubule motor activity, minus-end-directed, dynein intermediate chain binding, and actin filament binding, whereas the main GO terms for cell component category retrieved were proteinaceous extracellular matrix, extracellular matrix component, apical part of cell, basement membrane, collagen trimer, sarcomere, myofibril, contractile fiber part, myosin complex, and contractile fiber.

Differential alternative splicing in the RNA-Seq data from X-chromosome identified one significant alternative 5' splice site (A5SS) involving the *HSD17B10* gene and two events of skipped exon on *XIST* and *IDS* genes (**Supplementary Table S8**). No other events such as alternative 3' splice site (A3SS), mutually exclusive exons (MXE) and retained intron (RI) events were identified on X-chromosome.

For minimizing a possible bias associated with the use of only one matched control in the DE analysis, we included additional healthy control RNA-Seq samples obtained from SRA database. Expression comparison for genes within the deletion on group 1 (individual II.3 versus males) showed significant overexpression for *MCF2*, *SLC6A8*, *FUNDC2*, and *VBP1* genes in the individual II.3, whereas on group 2 (individual II.3 versus females), only *FUNDC2* gene showed significant values, being also overexpressed on individual II.3. No gene exhibited a significantly decreased value in both comparison groups (**Supplementary Table S9**). Besides, 167 from the 1,026 DE-genes identified in the first transcriptome-wide analysis (individual II.3 versus our matched control sample) were replicated (padj < 0.05), when we included more female controls (group 2) (**Supplementary Table S10**). The RNA-seq data (raw and processed files) for the individual II.3 and the matched control was deposited on GEO database (accession GSE141766).

## Discussion

Different strategies have evolved for equalizing X-chromosome expression between sexes in different organisms (Gelbart and Kuroda, 2009). In humans, XCI is characteristically incomplete, with a subset of 12–23% genes known to be also expressed from the Xi, called XCI escape genes (Carrel and Willard, 2005; Talebizadeh et al., 2006; Yasukochi et al., 2010; Cotton et al., 2013; Lister et al., 2013; Szelinger et al., 2014; Balaton et al., 2015; Cotton et al., 2015; Wainer-Katsir and Linial, 2016; Tukiainen et al., 2017). Human genes that escape from XCI tend not to be expressed to the same levels that are observed from the Xa (Balaton et al., 2015). Usually, an XCI escape gene shows ≥10% expression from the Xi allele compared with the Xa allele (Carrel and Willard, 2005). Some of the XCI escape genes are members of X-Y gene pairs with a paralogue on the Y chromosome, where they can have the same function as the X paralogue. Other XCI escape genes have lost their Y paralogue, or their Y paralogue has evolved a distinct, often testis-specific, role (Jegalian and Page, 1998; Deng et al., 2014) and highly conserved dosage-sensitive X/Y paralogs that escape from XCI in females are candidates for being responsible for embryo survival (Bellott et al., 2014).

Moreover, the number of XCI escape genes is bigger on the evolutionarily more recent strata of the X-chromosome (Ross et al., 2005; Balaton and Brown, 2016). Beyond the pseudoautosomal regions (PARs), one of the gene clusters expressed from Xi maps to the gene-rich region Xq28, where the expression level may reach 50% (Carrel and Willard, 2005). So, irrespective of whether mutations in XCI escape genes are located on the Xa or Xi, they could be detrimental (Fieremans et al., 2015).

In our study, we report on a female with a large Xq25-q28 deletion and extreme XCI skewing towards the altered paternal X-chromosome on blood and buccal mucosa. Regardless of the skewed XCI, the deletion forces the structural hemizygosis of XCI escape and variable escape genes. Within the Xq deletion, there are at least 16 fully XCI escape genes, 27 variable escape genes and a considerable number of additional genes with unknown XCI statuses [combined status described on (Tukiainen et al., 2017); **Supplementary Table S6**]. However, individual II.3 presented significant differential gene expression only for three blood-expressed genes spanning the deletion (*GPR112*, *SLC6A8*, *FUNDC2*) on transcriptome-wide analysis in comparison to the matched control. While *FUNDC2* is known to be subject to XCI, *GPR112* escapes XCI and *SLC6A8* has its XCI status yet unknown (Tukiainen et al., 2017). Surprisingly, *SLC6A8*, required for the uptake of creatine in muscles and brain (Fezai et al., 2014), and *FUNDC2*, that supports platelet survival *via* AKT signaling pathway (Ma et al., 2019), are overexpressed in individual II.3, in comparison to the matched control. The significant overexpression for *SLC6A8* and *FUNDC2* genes were corroborated by an additional DE analysis with male and female control samples obtained from the SRA database. Although *GPR112* did not demonstrate significant decreased expression on individual II.3 in such analysis, it could probably be due to methodological differences among the studies, concerning mainly RNA isolation and library preparation procedures. The observed equalized expression of most XCI escape and variable escape genes on Xa suggests that in this female occurs transcriptional upregulation of genes lost in the structurally abnormal X-chromosome, avoiding their functional haploinsufficiency.

X-chromosome is enriched in genes related to cognitive function (Zechner et al., 2001), and there is an excess of XCI escape genes associated with ID (Zhang et al., 2013), which is consistent with the presence of learning impairment in phenotypes associated to X-chromosome aneuploidies (Rooman et al., 2002). Moreover, the Xq25-q28 region is well known to be a hotspot for ID. Several deletions of the Xq25-q28 region in females with ID partly overlapping that seen in individual II.3 have been reported on the Decipher database (Firth et al., 2009). The consequences of such deletions can result in deregulation of the affected genes and may also reflect *trans*-acting effects on other chromosomal *loci* or even more global genomic alterations. Usually, the larger the deletion is, the more phenotypically detrimental it is, pointing to a cumulative effect. Notwithstanding, the hallmark in this female patient is the great extension of the deletion, including hotspot regions for ID and premature ovarian failure.

The proximal breakpoint of the Xq deletion according to array-CGH resides on *THOC2*, a gene subject to XCI that was previously associated to neurodevelopmental disorders in males (Kumar et al., 2015) and also in a female with a *de novo* missense variant (p.Tyr517Cys) and no available XCI status data (Kumar et al., 2018). The absence of significant differential expression for this gene suggests that individual II.3 was protected for presenting *THOC2* deleterious effects due to extreme XCI skewing. As recent transcriptome analysis suggests that XCI is generally uniform across human tissues (Tukiainen et al., 2017), we could speculate that the same X-chromosome was preferentially inactivated in different tissues other than blood and buccal mucosa.

Among the escape and variable escape genes within the Xq deletion, there are genes, whose mutations were previously associated with ID with clinical manifestation also in females, including *NAA10* (Gupta et al., 2019). Moreover, there are additional genes with fully/variable escape patterns or female bias profile related to essential biological functions or clinical conditions, such as *IKBKG*, associated to Incontinentia Pigmenti (**Supplementary Table S6**). Although there is still some divergence about the escape statuses of X-chromosome genes on the literature, our transcriptome results suggest that the individual II.3 compensated the expression, at transcription levels, for some blood-expressed XCI escape and variable escape genes within the deletion.

The unique apparent phenotype in individual II.3 is the presentation of POF at 34-years old. Two POF susceptibility regions have been identified: POF1 extends from Xq21-qter, including *FMR1* gene, whereas POF2 spreads from Xq13.3 to Xq21.1 (Lacombe et al., 2006). Indeed, terminal deletions at Xq were reported as part of a workup for infertility or POI and also in women screening for *FMR1* premutation (Yachelevich et al., 2011). Individual II.3 has no family history for POI/POF, despite the segregation of *FMR1* premutations in her family. Ovarian function in this female may be impaired by monosomy for genes required in double amount after X-chromosome reactivation for germ-cell development (Rossetti et al., 2004). Besides the deletion involving POF1 region, individual II.3 presented a significant differential expression for *POF1B* gene (log2 fold change value = -7,28; q value = 0,001), which is located at POF2 region (Xq21.1) and is proposed to escape from XCI. *POF1B* may act as an anti-apoptosis factor, slowing down the process of germ cell loss, so that *POF1B* loss of function mutations could lead to exaggerated germ cell apoptosis and POF (Lacombe et al., 2006). Recent advances have also demonstrated the importance of XCI escape genes in sexually dimorphic risk, particularly cancer (Balaton and Brown, 2016; Arnold and Disteche, 2018). Nonetheless, the COSMIC database did not disclose any oncogene among the differential expressed genes within the Xq deletion, yet a future clinical outcome cannot be eliminated. Besides *POF1B*, four autosomal differential expressed genes related to the term “premature ovarian failure” (HP:0008209) in the Human Phenotype Ontology (HPO) database were found and could have influenced in the only apparent phenotype of the patient: *CEP290* (log2 fold change value = 1,75; q value = 0,004), *HFM1* (log2 fold change value = -7,34; q value = 0,001), *STAG3* (log2 fold change value = -5,26; q value = 0,00001), and *NPHP4* (log2 fold change value = -8,65; q value = 0,0000003). Three of these autosomal genes (*CEP290*, *STAG3*, *NPHP4*) were replicated (padj < 0.05), when we added more female controls (group 2), exhibiting similar log2 fold change trends (positive or negative) (**Supplementary Table 10**). We should remark that although POF is the unique apparent phenotype in individual II.3, we cannot discard future clinical outcomes in the patient, mainly associated to the diseases, biological processes, molecular functions and cellular component enriched in the GO analysis for the global differentially expressed genes.

The most viable explanation for the absence of major clinical symptoms in the individual II.3 would be a post-zygotic mosaicism event, involving the concomitant presence of 46, XX, and Xq25-q28 deletion cells. Except for rs572013, all the other monoallelic blood-expressed SNPs within the deletion (rs859577, rs8965, rs1059703) are highly polymorphic in GnomAD Browser (Karczewski et al., 2019) with frequencies of 0.63, 0.52, and 0.68, respectively. The hemizygosity for these markers, in addition to the high metaphases, count in karyotype analysis, as well as blood and buccal mucosa hemizygosity concordance for microsatellite markers within the deletion argues against of the occurrence of mosaicism, at least in these different embryonic tissues. Altogether, the data also confirm the near to complete XCI skewing. Even that the skewed XCI may occur as a purely stochastic event and can vary between tissues and with age, XCI patterns in blood and buccal mucosa are accepted as a representative for the pattern in the brain and other tissues (Bittel et al., 2008).

The presence of an adjustable compensation mechanism on individual II.3 can demonstrate that gene-by-gene upregulation likely occurred on X-chromosome to reduce deleterious dosage imbalance. Indeed, two major types of X-chromosome dosage compensation can be recognized. One balances X-chromosome gene expression between sexes (achieved by XCI in mammals), and the other equalizes gene expression throughout the genome by changing the relative expression of X-linked genes versus autosomal genes and vice-versa (Disteche, 2016). While X-chromosome upregulation relative to autosomes is evident in flies, resulting from a combination of homeostatic gene-by-gene regulation and chromosome-wide regulation (Chen and Oliver, 2015), it is still controversial in mammals (Gupta et al., 2006; Nguyen and Disteche, 2006; Xiong et al., 2010; Deng et al., 2011; Chen and Zhang, 2015). In general, genes compensatory responses include (a) buffering or passive absorption of gene dose perturbation by inherent system properties, (b) feedback or gene-specific sensing and adjustment of levels, which can result in overexpression, and (c) feedforward responses representing systems, such as the male X-chromosome in Drosophila (Zhang et al., 2010; Disteche, 2016). These mechanisms may act individually or, more likely, in combination. Exploring experimentally these hypotheses/mechanisms in depth is, however, beyond the purpose of our study.

According to the recent literature, dosage upregulation in individual II.3 is presumably due to positive feedbacks mediated by enhanced transcription initiation, improved mRNA stability and epigenetic changes favoring expression, mechanisms already described in Drosophila, yeast, and mammals (Deng et al., 2013; Deng et al., 2014; Disteche, 2016). However, we could not exclude the participation of additional compensatory mechanisms at posttranscriptional level (e.g., modulation by non-coding RNAs as miRNAs and lnRNAs) and translational/posttranslational levels (e.g., increased ribosome density/decreased proteolysis) (Deng et al., 2014; Disteche, 2016). It should be noted that X-chromosome is particularly flexible to gene-by-gene dosage compensation, since increased transcription levels and RNA stability have independently evolved to upregulate individual X-linked genes after they lost their Y copy (Deng et al., 2013; Deng et al., 2014). Thus, X-linked transcripts appear to have a longer half-life than autosomal transcripts (Yin et al., 2009; Disteche, 2016) and gene-by-gene upregulation is known to differentially regulate subsets of ancestral and acquired X-linked genes to rich a balance with autosomes (Deng et al., 2013; Deng et al., 2014). Similar gene-by-gene compensation mechanisms were also described for other chromosomes. Imprinted genes in mice appear to be upregulated, alleviating deleterious effects at monoallelically expressed genes (Zaitoun et al., 2010). Although no imprinted gene has been identified on the human X-chromosome, there is an important overlap between XCI and such mechanism, since both are regulated by DNA methylation, histone modification, long non-coding RNAs and nuclear positioning. Furthermore, gene-by-gene downregulation was demonstrated in patients with Down syndrome (DS; MIM# 190685), in which 56% of the chromosome 21 transcripts are compensated for the gene-dosage effect, having mRNA levels similar to those of disomic genes (Aït Yahya-Graison et al., 2007).

One significant alternative 5' splice site (A5SS) involving the *HSD17B10* gene and two events of skipped exon on *XIST* and *IDS* genes alternative splicing were also identified in individual II.3. The role of these events is not clear, since they involve X-linked genes outside the deletion. Nonetheless, we cannot exclude that they might be associated with long-range effects of the aberration. Furthermore, it is worth mentioning that the presence of two different rare mutations (meiotic del Xq and *FMR1* expansion) in the same family is very unusual. The same paternal origin of the abnormal chromosomes led us to suspect that a common mechanism was responsible for the premutation allele in the mother (individual II.2) of the proband and the deleted X-chromosome in his aunt (individual II.3).

## Conclusions

Dosage compensation mechanisms associated with sex chromosomes demonstrate uncovered intricacies. Altogether, our data suggest that besides preferential inactivation of the structurally abnormal X-chromosome, an additional protective gene-by-gene mechanism occurs at the transcriptional level in the Xa to counterbalance detrimental effects of large Xq deletions, which can have high impact in genetic counseling. Further functional investigations in similar cases of females with large Xq deletions and no major detrimental phenotypes with high throughput technologies appraising gene expression combined to chromatin marks are needed to confirm the proposed upregulation compensatory mechanism in XCI escape/variable escape genes.

## Data Availability Statement

The datasets generated for this study can be found in the ClinVar (accession number SCV000897650) and GEO database (accession number GSE141766).

## Web Resources

The URLs for data presented herein are as follows: Catalogue Of Somatic Mutations In Cancer (COSMIC), https://cancer.sanger.ac.uk/cosmic (Tate et al., 2019). Decipher, https://decipher.sanger.ac.uk/ (Firth et al., 2009). FastQC, https://www.bioinformatics.babraham.ac.uk/projects/fastqc/ (Andrews, 2010). Gene Ontology Consortium, http://geneontology.org/ (Harris et al., 2004). Greenwood Genetic Center, Genes Involved in X-Linked Intellectual Disability by Order of Discovery (revised January 2020), https://www.ggc.org/pdf/Research/XLID_Table_Jan_2020.pdf. Genome Aggregation Database (gnomAD), (Karczewski et al., 2019). Human Disease Ontology, http://www.disease-ontology.org/ (Schriml et al., 2019). Multivariate Analysis of Transcript Splicing (rMATS), http://rnaseq-mats.sourceforge.net/ (Shen et al., 2014). TFCat, http://www.tfcat.ca/ (Fulton et al., 2009).

## Ethics Statement

The studies involving human participants were reviewed and approved by Rio de Janeiro State University. Written informed consent to participate in this study was provided by the participants' legal guardian/next of kin. Written informed consent was obtained from the individual(s) for the publication of any potentially identifiable images or data included in this article.

## Author Contributions

Conception and design: CS-R. Acquisition of data: CS-R, RB, EV, RP, AG, BA, JS, and VC. Analysis and interpretation: CS-R, RB, EV, RP, AG, FM, EM-A, and MP. Manuscript drafting: CS-R, RB, FM, EM-A, and MP. Obtained funding: CS-R and MP.

## Conflict of Interest

The authors declare that the research was conducted in the absence of any commercial or financial relationships that could be construed as a potential conflict of interest.

## References

[B1] Aït Yahya-Graison E. Aubert J. Dauphinot L. Rivals I. Prieur M. Golfier G. (2007). Classification of human chromosome 21 gene-expression variations in down syndrome: impact on disease phenotypes. Am. J. Hum. Genet. 81, 475–491. 10.1086/520000 17701894 PMC1950826

[B2] Anders S. Pyl P. T. Huber W. (2015). HTSeq–a python framework to work with high-throughput sequencing data. Bioinformatics 31, 166–169. 10.1093/bioinformatics/btu638 25260700 PMC4287950

[B3] Andrews S. (2010). FastQC - A quality control tool for high throughput sequence data., http://www.bioinformatics.babraham.ac.uk/projects/fastqc/

[B4] Arnold A. P. Disteche C. M. (2018). Sexual inequality in the cancer cell. Cancer Res. 78, 5504–5505. 10.1158/0008-5472.CAN-18-2219 30275051 PMC6204258

[B5] Balaton B. P. Brown C. J. (2016). Escape artists of the X chromosome. Trends Genet. 32, 348–359. 10.1016/j.tig.2016.03.007 27103486

[B6] Balaton B. P. Cotton A. M. Brown C. J. (2015). Derivation of consensus inactivation status for X-linked genes from genome-wide studies. Biol. Sex Differ. 6, 35. 10.1186/s13293-015-0053-7 26719789 PMC4696107

[B7] Bellott D. W. Hughes J. F. Skaletsky H. Brown L. G. Pyntikova T. Cho T.-J. (2014). Mammalian Y chromosomes retain widely expressed dosage-sensitive regulators. Nature 508, 494–499. 10.1038/nature13206 24759411 PMC4139287

[B8] Bittel D. C. Theodoro M. F. Kibiryeva N. Fischer W. Talebizadeh Z. Butler M. G. (2008). Comparison of X-chromosome inactivation patterns in multiple tissues from human females. J. Med. Genet. 45, 309–313. 10.1136/jmg.2007.055244 18156436 PMC5489244

[B9] Busque L. Paquette Y. Provost S. Roy D.-C. Levine R. L. Mollica L. (2009). Skewing of X-inactivation ratios in blood cells of aging women is confirmed by independent methodologies. Blood 113, 3472–3474. 10.1182/blood-2008-12-195677 19202126 PMC4729536

[B10] Carrel L. Willard H. F. (2005). X-inactivation profile reveals extensive variability in X-linked gene expression in females. Nature 434, 400–404. 10.1038/nature03479 15772666

[B11] Chen Z.-X. Oliver B. (2015). X chromosome and autosome dosage responses in drosophila melanogaster heads. G3 (Bethesda). 5, 1057–1063. 10.1534/g3.115.017632 25850426 PMC4478536

[B12] Chen X. Zhang J. (2015). No X-chromosome dosage compensation in human proteomes. Mol. Biol. Evol. 32, 1456–1460. 10.1093/molbev/msv036 25697342 PMC4462673

[B13] Chen E. Y. Tan C. M. Kou Y. Duan Q. Wang Z. Meirelles G. (2013). Enrichr: interactive and collaborative HTML5 gene list enrichment analysis tool. BMC Bioinf. 14, 128. 10.1186/1471-2105-14-128 PMC3637064 23586463

[B14] Cotton A. M. Ge B. Light N. Adoue V. Pastinen T. Brown C. J. (2013). Analysis of expressed SNPs identifies variable extents of expression from the human inactive X chromosome. Genome Biol. 14, R122. 10.1186/gb-2013-14-11-r122 24176135 PMC4053723

[B15] Cotton A. M. Price E. M. Jones M. J. Balaton B. P. Kobor M. S. Brown C. J. (2015). Landscape of DNA methylation on the X chromosome reflects CpG density, functional chromatin state and X-chromosome inactivation. Hum. Mol. Genet. 24, 1528–1539. 10.1093/hmg/ddu564 25381334 PMC4381753

[B16] de Araujo L. S. Vaas L. A. I. Ribeiro-Alves M. Geffers R. Mello F. C. Q. de Almeida A. S. (2016). transcriptomic biomarkers for tuberculosis: evaluation of DOCK9. EPHA4, and NPC2 mRNA expression in peripheral blood. Front. Microbiol. 7, 1586. 10.3389/fmicb.2016.01586 27826286 PMC5078140

[B17] Deng X. Hiatt J. B. Nguyen D. K. Ercan S. Sturgill D. Hillier L. W. (2011). Evidence for compensatory upregulation of expressed X-linked genes in mammals, Caenorhabditis elegans and Drosophila melanogaster. Nat. Genet. 43, 1179–1185. 10.1038/ng.948 22019781 PMC3576853

[B18] Deng X. Berletch J. B. Ma W. Nguyen D. K. Hiatt J. B. Noble W. S. (2013). Mammalian X upregulation is associated with enhanced transcription initiation, RNA half-life, and MOF-mediated H4K16 acetylation. Dev. Cell 25, 55–68. 10.1016/j.devcel.2013.01.028 23523075 PMC3662796

[B19] Deng X. Berletch J. B. Nguyen D. K. Disteche C. M. (2014). X chromosome regulation: diverse patterns in development, tissues and disease. Nat. Rev. Genet. 15, 367–378. 10.1038/nrg3687 24733023 PMC4117651

[B20] Disteche C. M. (1999). Escapees on the X chromosome. Proc. Natl. Acad. Sci. 96, 14180–14182. 10.1073/pnas.96.25.14180 10588671 PMC33938

[B21] Disteche C. M. (2016). Dosage compensation of the sex chromosomes and autosomes. Semin. Cell Dev. Biol. 56, 9–18. 10.1016/j.semcdb.2016.04.013 27112542 PMC4955796

[B22] Dobin A. Davis C. A. Schlesinger F. Drenkow J. Zaleski C. Jha S. (2013). STAR: ultrafast universal RNA-seq aligner. Bioinformatics 29, 15–21. 10.1093/bioinformatics/bts635 23104886 PMC3530905

[B23] Fezai M. Elvira B. Borras J. Ben-Attia M. Hoseinzadeh Z. Lang F. (2014). Negative regulation of the creatine transporter SLC6A8 by SPAK and OSR1. Kidney Blood Press Res. 39, 546–554. 10.1159/000368465 25531585

[B24] Fieremans N. Van Esch H. de Ravel T. Van Driessche J. Belet S. Bauters M. (2015). Microdeletion of the escape genes KDM5C and IQSEC2 in a girl with severe intellectual disability and autistic features. Eur. J. Med. Genet. 58, 324–327. 10.1016/j.ejmg.2015.03.003 25858702

[B25] Fieremans N. Van Esch H. Holvoet M. Van Goethem G. Devriendt K. Rosello M. (2016). Identification of intellectual disability genes in female patients with a Skewed X-inactivation pattern. Hum. Mutat. 37, 804–811. 10.1002/humu.23012 27159028 PMC4940233

[B26] Firth H. V. Richards S. M. Bevan A. P. Clayton S. Corpas M. Rajan D. (2009). DECIPHER: Database of Chromosomal Imbalance and Phenotype in Humans Using Ensembl Resources. Am. J. Hum. Genet. 84, 524–533. 10.1016/j.ajhg.2009.03.010 19344873 PMC2667985

[B27] Fulton D. L. Sundararajan S. Badis G. Hughes T. R. Wasserman W. W. Roach J. C. (2009). TFCat: the curated catalog of mouse and human transcription factors. Genome Biol. 10, R29 10.1186/gb-2009-10-3-r29 19284633 PMC2691000

[B28] Gelbart M. E. Kuroda M. I. (2009). Drosophila dosage compensation: a complex voyage to the X chromosome. Development 136, 1399–1410. 10.1242/dev.029645 19363150 PMC2674252

[B29] Gonçalves T. F. dos Santos J. M. Gonçalves A. P. Tassone F. Mendoza-Morales G. Ribeiro M. G. (2016). Finding FMR1 mosaicism in Fragile X syndrome. Expert Rev. Mol. Diagn. 16, 501–507. 10.1586/14737159.2016.1135739 26716517 PMC4956488

[B30] Gupta V. Parisi M. Sturgill D. Nuttall R. Doctolero M. Dudko O. K. (2006). Global analysis of X-chromosome dosage compensation. J. Biol. 5, 3. 10.1186/jbiol30 16507155 PMC1414069

[B31] Gupta A. S. Saif,.H. Al Lent J. M. Couser N. L. (2019). Ocular manifestations of the NAA10 -related syndrome. Case Rep. Genet. 2019, 1–6. 10.1155/2019/8492965 PMC6476065 31093388

[B32] Harris M. A. Clark J. Ireland A. Lomax J. Ashburner M. Foulger R. (2004). The Gene Ontology (GO) database and informatics resource. Nucleic Acids Res. 32, D258–261. 10.1093/nar/gkh036 14681407 PMC308770

[B33] Hurst L. D. Ghanbarian A. T. Forrest A. R. R. Huminiecki L. (2015). The constrained maximal expression level owing to haploidy shapes gene content on the mammalian X chromosome. PloS Biol. 13, e1002315. 10.1371/journal.pbio.1002315 26685068 PMC4686125

[B34] Jegalian K. Page D. C. (1998). A proposed path by which genes common to mammalian X and Y chromosomes evolve to become X inactivated. Nature 394, 776–780. 10.1038/29522 9723615

[B35] Karczewski K. J. Francioli L. C. Tiao G. Cummings B. B. Alföldi J. Wang Q. (2019). Variation across 141,456 human exomes and genomes reveals the spectrum of loss-of-function intolerance across human protein-coding genes. bioRxiv. 531210. 10.1101/531210

[B36] Kuleshov M. V. Jones M. R. Rouillard A. D. Fernandez N. F. Duan Q. Wang Z. (2016). Enrichr: a comprehensive gene set enrichment analysis web server 2016 update. Nucleic Acids Res. 44, W90–W97. 10.1093/nar/gkw377 27141961 PMC4987924

[B37] Kumar R. Corbett M. A. van Bon B. W. M. Woenig J. A. Weir L. Douglas E. (2015). THOC2 mutations implicate mRNA-export pathway in X-linked intellectual disability. Am. J. Hum. Genet. 97, 302–310. 10.1016/j.ajhg.2015.05.021 26166480 PMC4573269

[B38] Kumar R. Gardner A. Homan C. C. Douglas E. Mefford H. Wieczorek D. (2018). Severe neurocognitive and growth disorders due to variation in THOC2, an essential component of nuclear mRNA export machinery. Hum. Mutat. 39, 1126–1138. 10.1002/humu.23557 29851191 PMC6481655

[B39] Lacombe A. Lee H. Zahed L. Choucair M. Muller J.-M. Nelson S. F. (2006). Disruption of POF1B binding to nonmuscle actin filaments is associated with premature ovarian failure. Am. J. Hum. Genet. 79, 113–119. 10.1086/505406 16773570 PMC1474115

[B40] Lister R. Mukamel E. A. Nery J. R. Urich M. Puddifoot C. A. Johnson N. D. (2013). Global epigenomic reconfiguration during mammalian brain development. Science (80-.)341, 1237905–1237905. 10.1126/science.1237905 PMC3785061 23828890

[B41] Ma Q. Zhu C. Zhang W. Ta N. Zhang R. Liu L. (2019). Mitochondrial PIP3-binding protein FUNDC2 supports platelet survival *via* AKT signaling pathway. Cell Death Differ. 26, 321–331. 10.1038/s41418-018-0121-8 29786068 PMC6329745

[B42] Machado F. B. Machado F. B. Faria M. A. Lovatel V. L. Alves da Silva A. F. Radic C. P. (2014). 5meCpG epigenetic marks neighboring a primate-conserved core promoter short tandem repeat indicate X-chromosome inactivation. PloS One 9, e103714. 10.1371/journal.pone.0103714 25078280 PMC4117532

[B43] Martin M. (2011). Cutadapt removes adapter sequences from high-throughput sequencing reads. EMBnet.journal 17, 3. 10.14806/ej.17.1.200

[B44] McKenna A. Hanna M. Banks E. Sivachenko A. Cibulskis K. Kernytsky A. (2010). The genome analysis toolkit: A MapReduce framework for analyzing next-generation DNA sequencing data. Genome Res. 20, 1297–1303. 10.1101/gr.107524.110 20644199 PMC2928508

[B45] McMahon A. Monk M. (1983). X -chromosome activity in female mouse embryos heterozygous for Pgk-1 and Searle's translocation, T(X; 16) 16H. Genet. Res. 41, 69–83. 10.1017/S0016672300021078 6840549

[B46] Morey C. Avner P. (2011). The Demoiselle of X-inactivation: 50 years old and as trendy and mesmerising as ever. PloS Genet. 7, e1002212. 10.1371/journal.pgen.1002212 21811421 PMC3141017

[B47] Mortazavi A. Williams B. A. McCue K. Schaeffer L. Wold B. (2008). Mapping and quantifying mammalian transcriptomes by RNA-Seq. Nat. Methods 5, 621–628. 10.1038/nmeth.1226 18516045 PMC13303166

[B48] Nguyen D. K. Disteche C. M. (2006). Dosage compensation of the active X chromosome in mammals. Nat. Genet. 38, 47–53. 10.1038/ng1705 16341221

[B49] Ogilvie C. M. Donaghue C. Fox S. P. Docherty Z. Mann K. (2005). Rapid prenatal diagnosis of aneuploidy using quantitative fluorescence-PCR (QF-PCR). J. Histochem. Cytochem. 53, 285–288. 10.1369/jhc.4B6409.2005 15750003

[B50] Reijnders M. R. F. Zachariadis V. Latour B. Jolly L. Mancini G. M. Pfundt R. (2016). De novo loss-of-function mutations in USP9X cause a female-specific recognizable syndrome with developmental delay and congenital malformations. Am. J. Hum. Genet. 98, 373–381. 10.1016/j.ajhg.2015.12.015 26833328 PMC4746365

[B51] Robinson M. D. McCarthy D. J. Smyth G. K. (2010). edgeR: a Bioconductor package for differential expression analysis of digital gene expression data. Bioinformatics 26, 139–140. 10.1093/bioinformatics/btp616 19910308 PMC2796818

[B52] Robinson J. T. Thorvaldsdóttir H. Winckler W. Guttman M. Lander E. S. Getz G. (2011). Integrative genomics viewer. Nat. Biotechnol. 29, 24–26 10.1038/nbt.1754 21221095 PMC3346182

[B53] Rooman R. P. A. Van Driessche K. Du Caju M. V. L. (2002). Growth and ovarian function in girls with 48,XXXX karyotype–patient report and review of the literature. J. Pediatr. Endocrinol. Metab. 15, 1051–1055. 10.1515/JPEM.2002.15.7.1051 12199336

[B54] Ross M. T. Grafham D. V. Coffey A. J. Scherer S. McLay K. Muzny D. (2005). The DNA sequence of the human X chromosome. Nature 434, 325–337. 10.1038/nature03440 15772651 PMC2665286

[B55] Rossetti F. Rizzolio F. Pramparo T. Sala C. Bione S. Bernardi F. (2004). A susceptibility gene for premature ovarian failure (POF) maps to proximal Xq28. Eur. J. Hum. Genet. 12, 829–834. 10.1038/sj.ejhg.5201186 15280899

[B56] Santos-Rebouças C. B. de Almeida L. G. Belet S. dos Santos S. R. Ribeiro M. G. da Silva A. F. A. (2015). Novel microduplications at Xp11.22 including HUWE1: clinical and molecular insights into these genomic rearrangements associated with intellectual disability. J. Hum. Genet. 60, 207–211. 10.1038/jhg.2015.1 25652354

[B57] Schriml L. M. Mitraka E. Munro J. Tauber B. Schor M. Nickle L. (2019). Human Disease Ontology 2018 update: classification, content and workflow expansion. Nucleic Acids Res. 47, D955–D962. 10.1093/nar/gky1032 30407550 PMC6323977

[B58] Schmidt M. Du Sart D. Kalitsis P. Fraser N. Leversha M. Voullaire L. (1991). X chromosome inactivation in fibroblasts of mentally retarded female carriers of the fragile site Xq27.3: application of the probe M27β to evaluate X inactivation status. Am. J. Med. Genet. 38, 411–415. 10.1002/ajmg.1320380252 1673316

[B59] Shen S. Park J. W. Lu Z. Lin L. Henry M. D. Wu Y. N. (2014). rMATS: robust and flexible detection of differential alternative splicing from replicate RNA-Seq data. Proc. Natl. Acad. Sci. 111, E5593–E5601. 10.1073/pnas.1419161111 25480548 PMC4280593

[B60] Snijders Blok L. Madsen E. Juusola J. Gilissen C. Baralle D. Reijnders M. R. F. (2015). Mutations in DDX3X are a common cause of unexplained intellectual disability with gender-specific effects on wnt signaling. Am. J. Hum. Genet. 97, 343–352. 10.1016/j.ajhg.2015.07.004 26235985 PMC4573244

[B61] Szelinger S. Malenica I. Corneveaux J. J. Siniard A. L. Kurdoglu A. A. Ramsey K. M. (2014). Characterization of X chromosome inactivation using integrated analysis of whole-exome and mRNA sequencing. PloS One 9, e113036. 10.1371/journal.pone.0113036 25503791 PMC4264736

[B62] Talebizadeh Z. Simon S. D. Butler M. G. (2006). X chromosome gene expression in human tissues: male and female comparisons. Genomics 88, 675–681. 10.1016/j.ygeno.2006.07.016 16949791 PMC7374763

[B63] Tate J. G. Bamford S. Jubb H. C. Sondka Z. Beare D. M. Bindal N. (2019). COSMIC: the catalogue of somatic mutations in cancer. Nucleic Acids Res. 47, D941–D947. 10.1093/nar/gky1015 30371878 PMC6323903

[B64] Tsuchiya K. D. Greally J. M. Yi Y. Noel K. P. Truong J. P. Disteche C. M. (2004). Comparative sequence and X-inactivation analyses of a domain of escape in human Xp11.2 and the conserved segment in mouse. Genome Res. 14, 1275–1284. 10.1101/gr.2575904 15197169 PMC442142

[B65] Tukiainen T. Villani A.-C. Yen A. Rivas M. A. Marshall J. L. Satija R. (2017). Landscape of X chromosome inactivation across human tissues. Nature 550, 244–248. 10.1038/nature24265 29022598 PMC5685192

[B66] Wainer-Katsir K. Linial M. (2016). Single cell expression data reveal human genes that escape X-chromosome inactivation. bioRxiv, 079830. 10.1101/079830

[B67] Wang K. Li M. Hakonarson H. (2010). ANNOVAR: functional annotation of genetic variants from high-throughput sequencing data. Nucleic Acids Res. 38, e164–e164. 10.1093/nar/gkq603 20601685 PMC2938201

[B68] Xiong Y. Chen X. Chen Z. Wang X. Shi S. Wang X. (2010). RNA sequencing shows no dosage compensation of the active X-chromosome. Nat. Genet. 42, 1043–1047. 10.1038/ng.711 21102464

[B69] Yachelevich N. Gittler J. K. Klugman S. Feldman B. Martin J. Brooks S. S. (2011). Terminal deletions of the long arm of chromosome X that include the FMR1 gene in female patients: a case series. Am. J. Med. Genet. Part A 155, 870–874. 10.1002/ajmg.a.33936 21595002

[B70] Yasukochi Y. Maruyama O. Mahajan M. C. Padden C. Euskirchen G. M. Schulz V. (2010). X chromosome-wide analyses of genomic DNA methylation states and gene expression in male and female neutrophils. Proc. Natl. Acad. Sci. 107, 3704–3709. 10.1073/pnas.0914812107 20133578 PMC2840519

[B71] Yin S. Deng W. Zheng H. Zhang Z. Hu L. Kong X. (2009). Evidence that the nonsense-mediated mRNA decay pathway participates in X chromosome dosage compensation in mammals. Biochem. Biophys. Res. Commun. 383, 378–382. 10.1016/j.bbrc.2009.04.021 19364502

[B72] Yu G. Wang L.-G. Han Y. He Q.-Y. (2012). clusterProfiler: an R package for comparing biological themes among gene clusters. *Omi* . A J. Integr. Biol. 16, 284–287. 10.1089/omi.2011.0118 PMC3339379 22455463

[B73] Zaitoun I. Downs K. M. Rosa G. J. M. Khatib H. (2010). Upregulation of imprinted genes in mice: an insight into the intensity of gene expression and the evolution of genomic imprinting. Epigenetics 5, 149–158. 10.4161/epi.5.2.11081 20168089 PMC3020655

[B74] Zechner U. Wilda M. Kehrer-Sawatzki H. Vogel W. Fundele R. Hameister H. (2001). A high density of X-linked genes for general cognitive ability: a run-away process shaping human evolution? Trends Genet. 17, 697–701. 10.1016/S0168-9525(01)02446-5 11718922

[B75] Zhang Y. Malone J. H. Powell S. K. Periwal V. Spana E. MacAlpine D. M. (2010). Expression in aneuploid drosophila S2 Cells. PloS Biol. 8, e1000320. 10.1371/journal.pbio.1000320 20186269 PMC2826376

[B76] Zhang Y. Castillo-Morales A. Jiang M. Zhu Y. Hu L. Urrutia A. O. (2013). Genes that escape X-inactivation in humans have high intraspecific variability in expression, are associated with mental impairment but are not slow evolving. Mol. Biol. Evol. 30, 2588–2601. 10.1093/molbev/mSM148 24023392 PMC3840307

