# Supplementary material for: Skewed X-Chromosome Inactivation and Compensatory Upregulation of Escape Genes Precludes Major Clinical Symptoms in a Female With a Large Xq Deletion
Source: Front Genet. 2020 Mar 4;11:101. doi: 10.3389/fgene.2020.00101 (PMC7064548; doi:10.3389/fgene.2020.00101)
Supplement: Supplementary file 1 [file Presentation_1.pptx]

## Slide 1
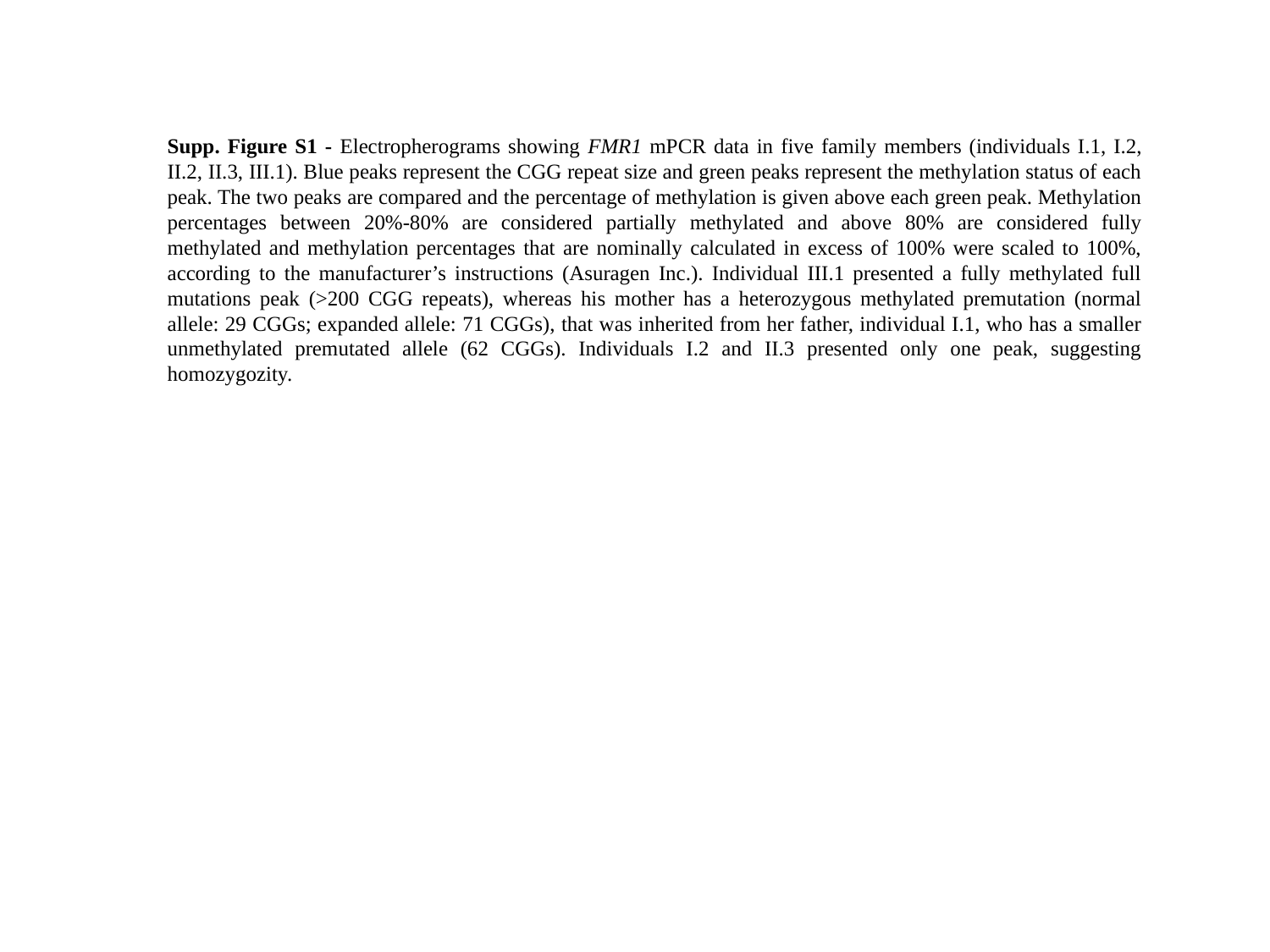

Supp. Figure S1 - Electropherograms showing FMR1 mPCR data in five family members (individuals I.1, I.2, II.2, II.3, III.1). Blue peaks represent the CGG repeat size and green peaks represent the methylation status of each peak. The two peaks are compared and the percentage of methylation is given above each green peak. Methylation percentages between 20%-80% are considered partially methylated and above 80% are considered fully methylated and methylation percentages that are nominally calculated in excess of 100% were scaled to 100%, according to the manufacturer’s instructions (Asuragen Inc.). Individual III.1 presented a fully methylated full mutations peak (>200 CGG repeats), whereas his mother has a heterozygous methylated premutation (normal allele: 29 CGGs; expanded allele: 71 CGGs), that was inherited from her father, individual I.1, who has a smaller unmethylated premutated allele (62 CGGs). Individuals I.2 and II.3 presented only one peak, suggesting homozygozity.

## Slide 2
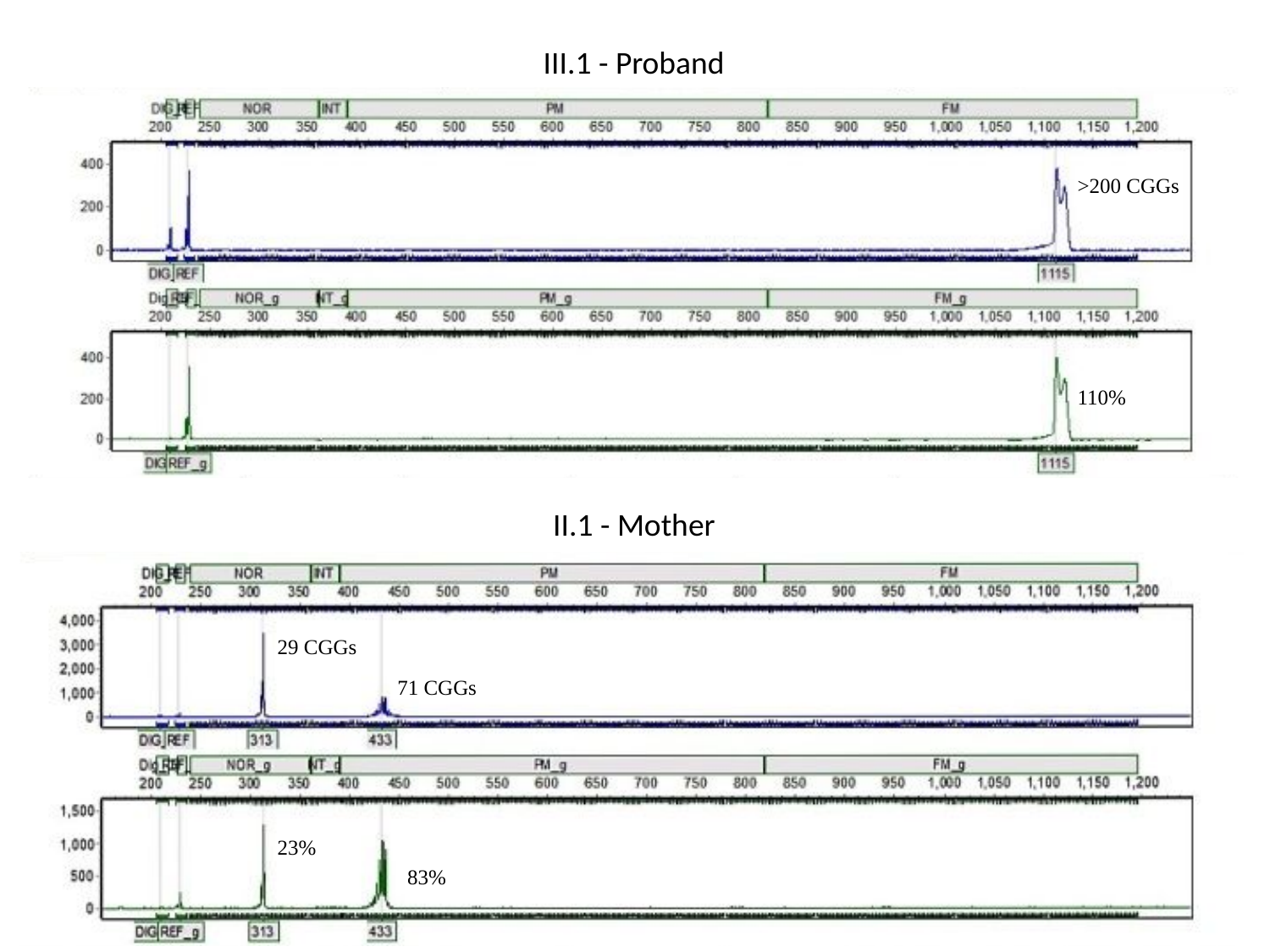

III.1 - Proband
>200 CGGs
II.1 - Mother
29 CGGs
71 CGGs
23%
83%
110%

## Slide 3
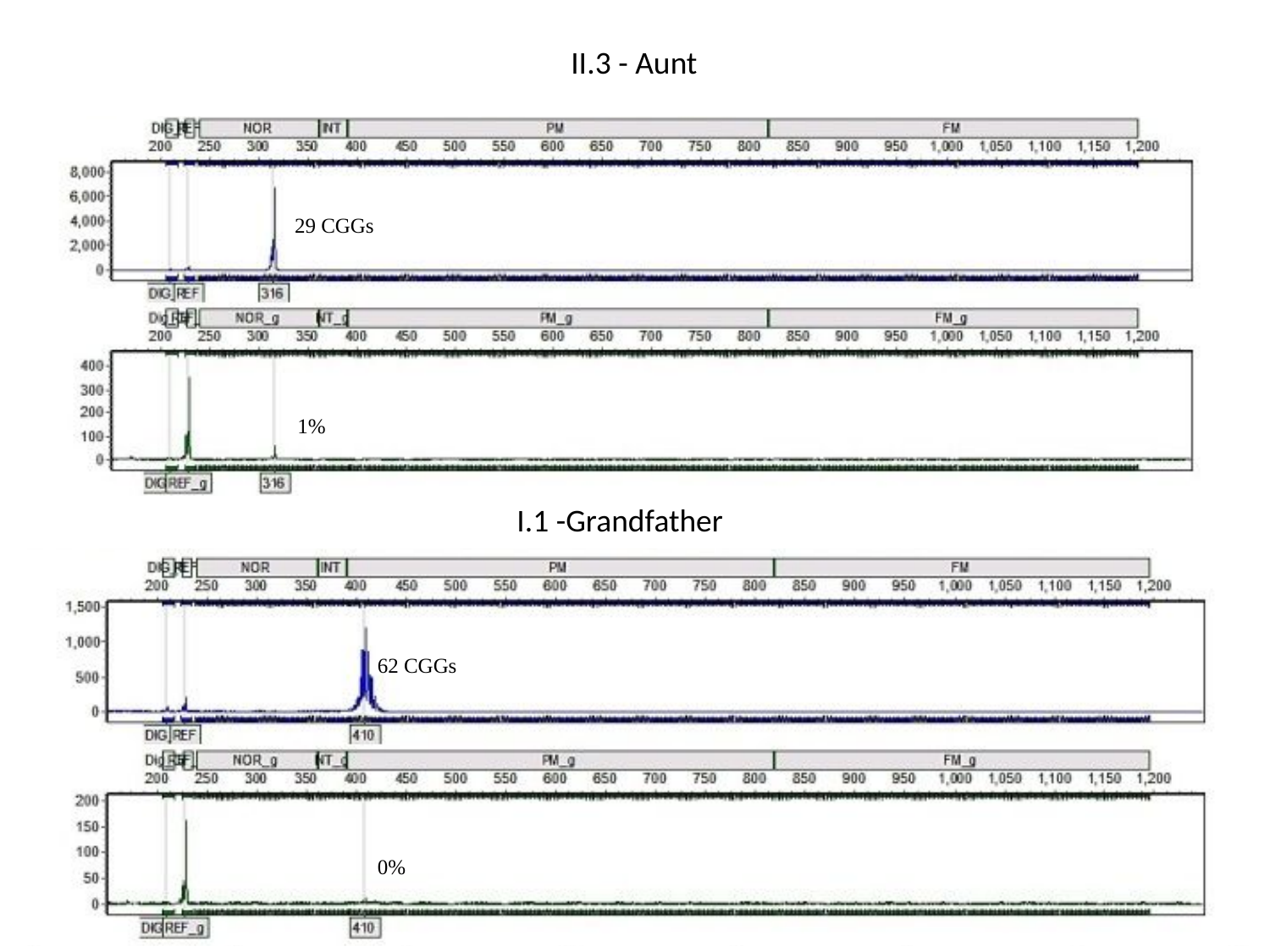

II.3 - Aunt
29 CGGs
1%
I.1 -Grandfather
62 CGGs
0%

## Slide 4
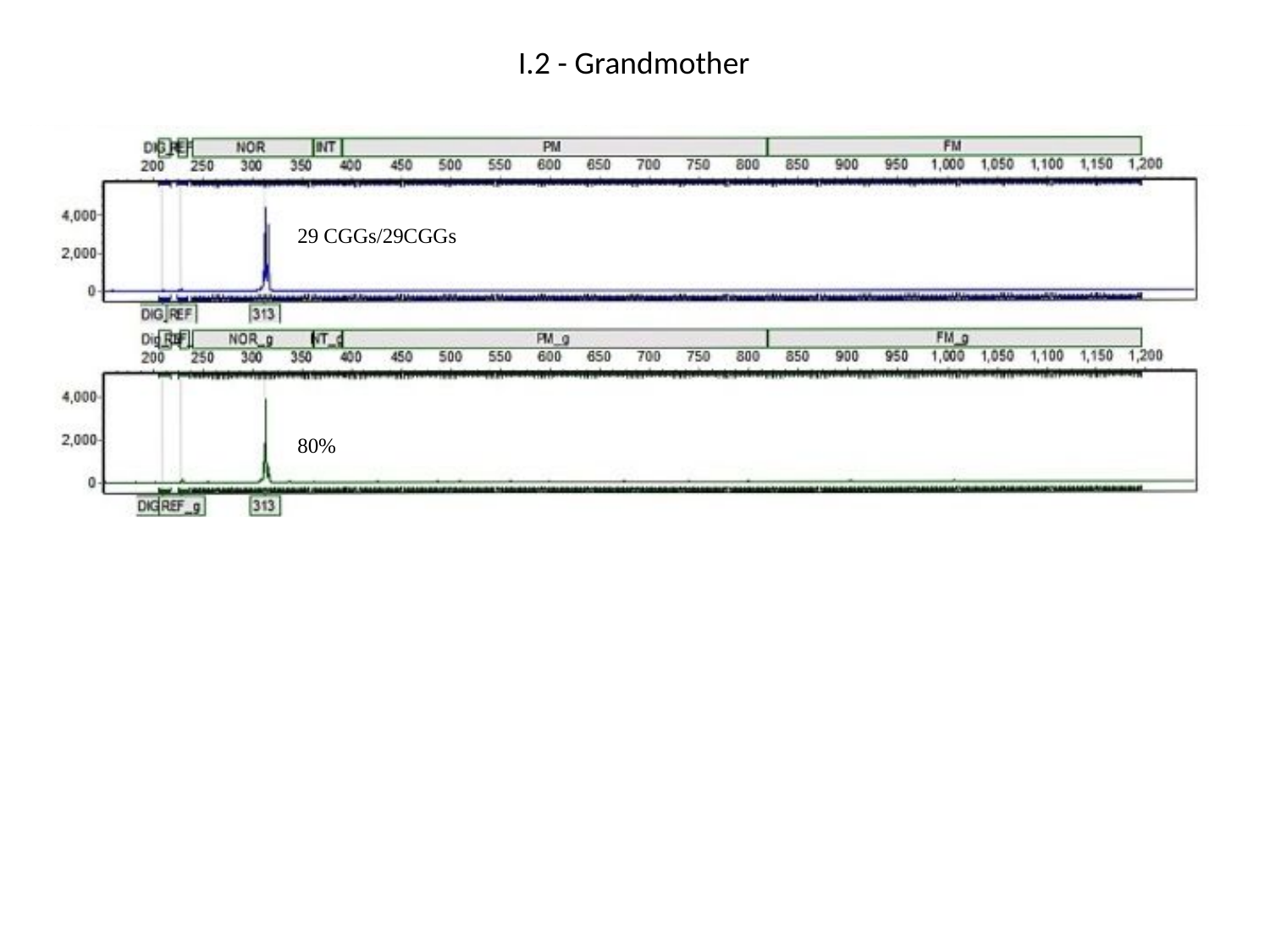

I.2 - Grandmother
29 CGGs/29CGGs
80%

## Slide 5
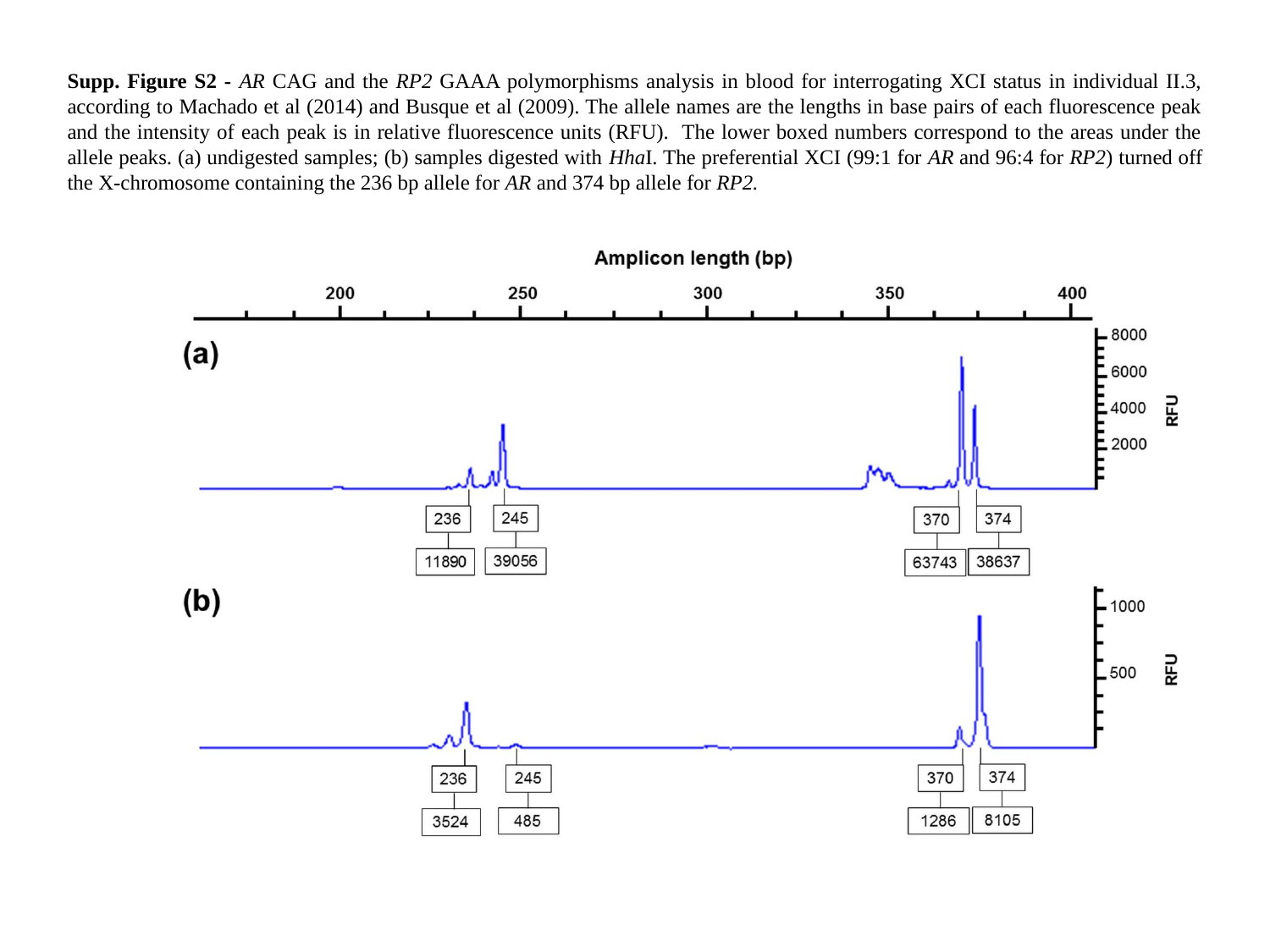

Supp. Figure S2 - AR CAG and the RP2 GAAA polymorphisms analysis in blood for interrogating XCI status in individual II.3, according to Machado et al (2014) and Busque et al (2009). The allele names are the lengths in base pairs of each fluorescence peak and the intensity of each peak is in relative fluorescence units (RFU). The lower boxed numbers correspond to the areas under the allele peaks. (a) undigested samples; (b) samples digested with HhaI. The preferential XCI (99:1 for AR and 96:4 for RP2) turned off the X-chromosome containing the 236 bp allele for AR and 374 bp allele for RP2.

## Slide 6
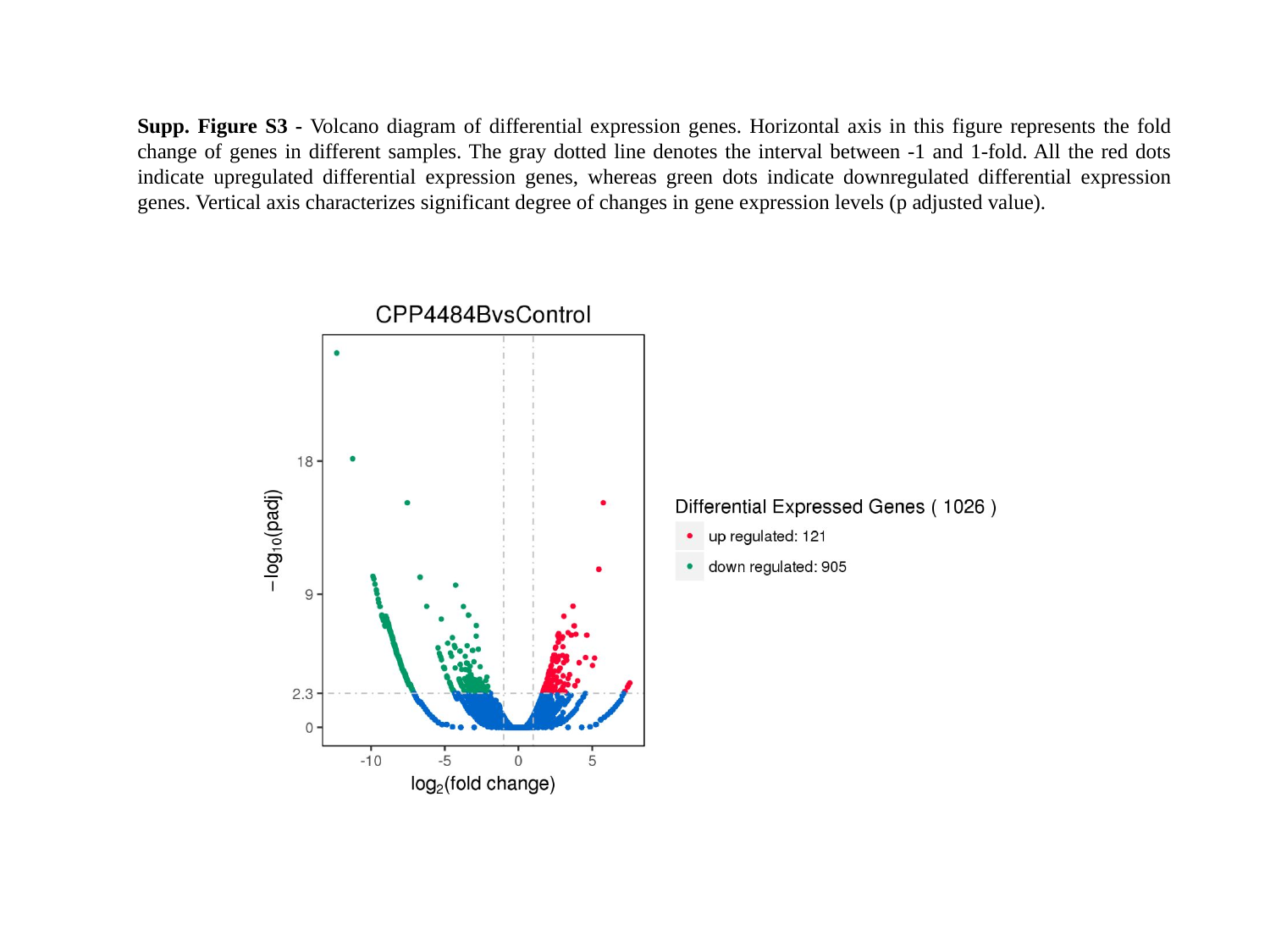

Supp. Figure S3 - Volcano diagram of differential expression genes. Horizontal axis in this figure represents the fold change of genes in different samples. The gray dotted line denotes the interval between -1 and 1-fold. All the red dots indicate upregulated differential expression genes, whereas green dots indicate downregulated differential expression genes. Vertical axis characterizes significant degree of changes in gene expression levels (p adjusted value).

## Slide 7
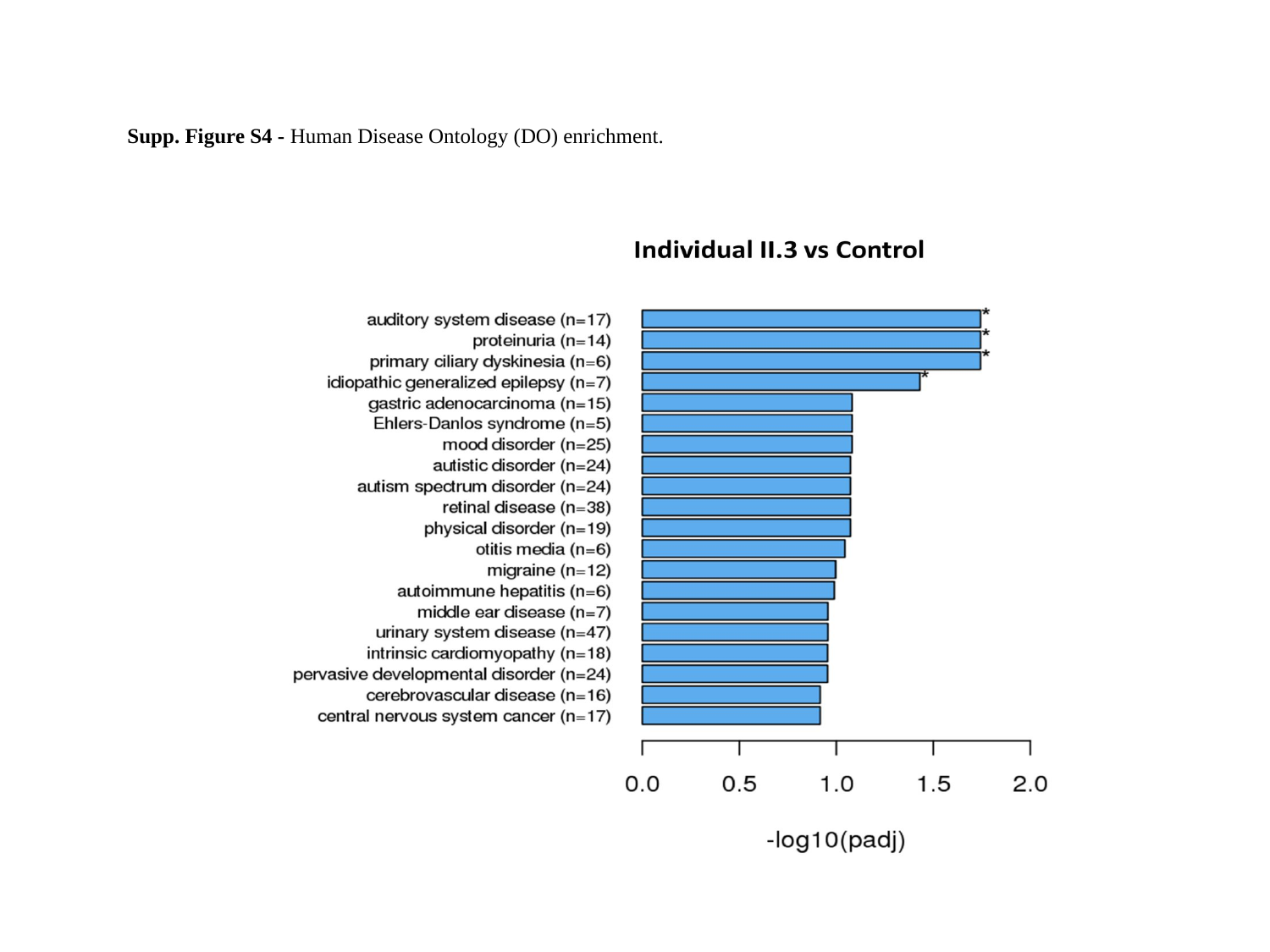

Supp. Figure S4 - Human Disease Ontology (DO) enrichment.

## Slide 8
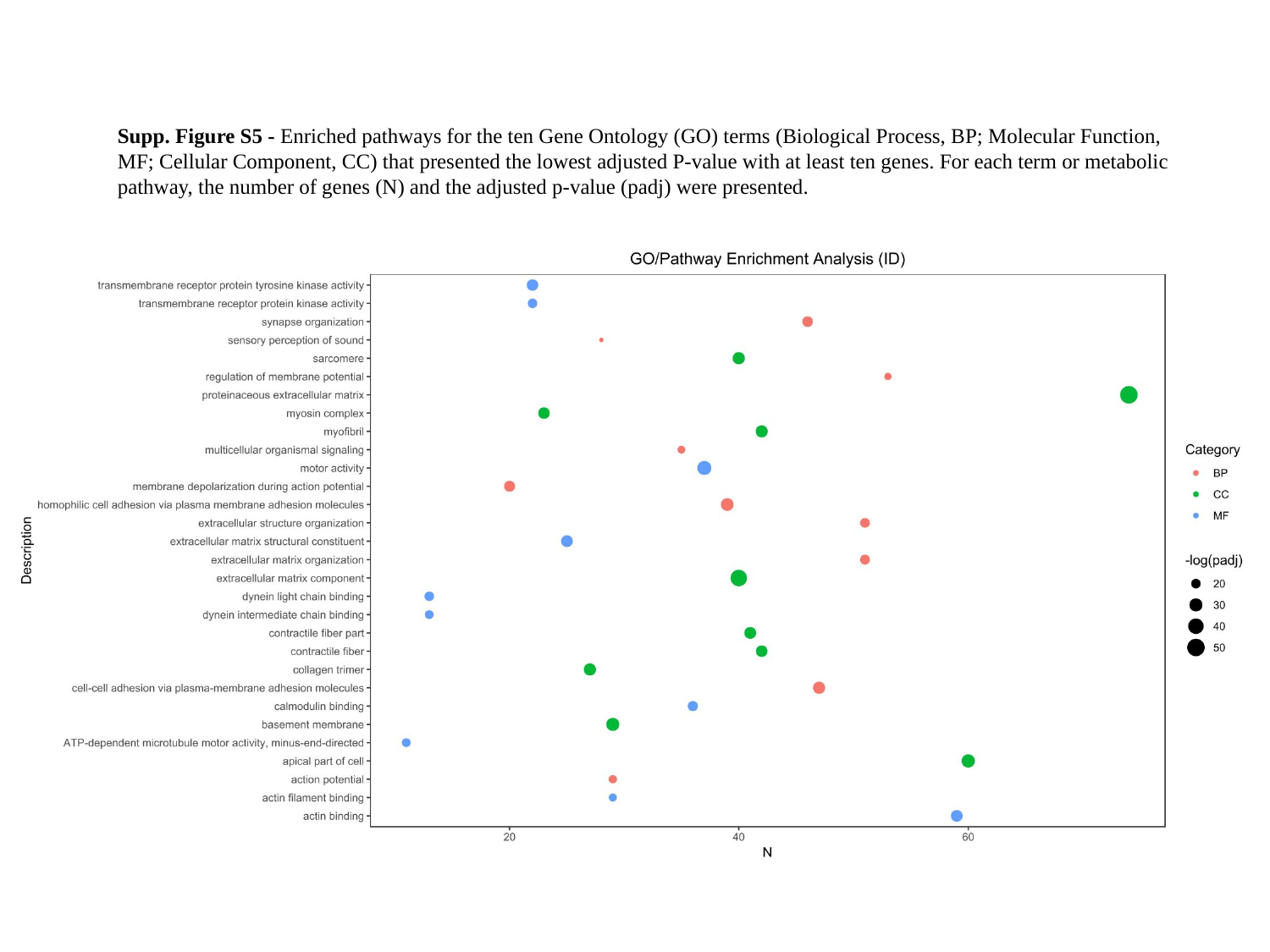

Supp. Figure S5 - Enriched pathways for the ten Gene Ontology (GO) terms (Biological Process, BP; Molecular Function, MF; Cellular Component, CC) that presented the lowest adjusted P-value with at least ten genes. For each term or metabolic pathway, the number of genes (N) and the adjusted p-value (padj) were presented.
