# Supplementary material for: Skewed X-Chromosome Inactivation and Compensatory Upregulation of Escape Genes Precludes Major Clinical Symptoms in a Female With a Large Xq Deletion
Source: Front Genet. 2020 Mar 4;11:101. doi: 10.3389/fgene.2020.00101 (PMC7064548; doi:10.3389/fgene.2020.00101)
Supplement: Supplementary file 2 [file Table_1.docx]

**Supp. Table S1-** Microsatellite markers analysis in the studied family. Segregation analysis distinguishes the paternal origin of the preferentially skewed Xi present in the individual II.3. Xi is identified based on the 236 bp *AR* allele and the 374 bp *RP2* allele.

| **Marker** | **Genomic Position (hg19)** | **I.1**  **grandfather** | |  | **I.2**  **grandmother** | |  | **II.2 mother** | |  |  | **II.3**  **aunt** | |  |  | **III.1 proband** | |
| --- | --- | --- | --- | --- | --- | --- | --- | --- | --- | --- | --- | --- | --- | --- | --- | --- | --- |
|  |  |  |  |  |  |  |  |  |  |  |  |  |  |  |  |  |  |
| DXS1283E | chrX:7867443-7867755 | **319** |  |  | **317** | **317** |  | **319** | **317** |  |  | **319** | **317** |  |  | **319** |  |
| *RP2* | chrX:46695746-46696127 | **374** |  |  | **370** | **350** |  | **374** | **370** |  | Xi | **374** | **370** | **Xa** |  | **370** |  |
| *AR* | chrX:66765076-66765319 | **236** |  |  | **245** | **218** |  | **236** | **245** |  | Xi | **236** | **245** | **Xa** |  | **245** |  |
| DXS981 | chrX:68197359-68197602 | **247** |  |  | **234** | **247** |  | **247** | **234** |  |  | **247** | **234** |  |  | **234** |  |
| DXS1187 | chrX:131033176-131033324 | **154** |  |  | **150** | **145** |  | **154** | **145** |  |  | **del** | **150** |  |  | **154** |  |
| XHPRT | chrX:133615405-133615691 | **284** |  |  | **292** | **281** |  | **284** | **281** |  |  | **del** | **292** |  |  | **284** |  |
| P39 | chrX:152632108-152632267 | **153** |  |  | **153** | **157** |  | **153** | **157** |  |  | **del** | **153** |  |  | **153** |  |
| X22 | chrX:154981543-154981768/  chrY:59084549-59084774 | **239** | **207** |  | **239** | **234** |  | **239** | **234** |  |  | **del** | **239** |  |  | **239** | **207** |
