# Supplementary material for: Skewed X-Chromosome Inactivation and Compensatory Upregulation of Escape Genes Precludes Major Clinical Symptoms in a Female With a Large Xq Deletion
Source: Front Genet. 2020 Mar 4;11:101. doi: 10.3389/fgene.2020.00101 (PMC7064548; doi:10.3389/fgene.2020.00101)
Supplement: Supplementary file 3 [file Table_2.docx]

**Supp. Table S2-** XCI patterns for *AR* and *RP2* markers in blood and buccal mucosa from individual II.3.

| **Tissue** | ***AR* marker** | | ***RP2* marker** | |
| --- | --- | --- | --- | --- |
|  | **Fragments lenght** | **XCI pattern** | **Fragments lenght** | **XCI pattern** |
| **Blood** | 236/245 | 99:1 | 370/374 | 4:96 |
| **Buccal mucosa** | 236/245 | 90:10 | 370/374 | 12:88 |

Underlined alleles represent the Xi.
