# Supplementary material for: Skewed X-Chromosome Inactivation and Compensatory Upregulation of Escape Genes Precludes Major Clinical Symptoms in a Female With a Large Xq Deletion
Source: Front Genet. 2020 Mar 4;11:101. doi: 10.3389/fgene.2020.00101 (PMC7064548; doi:10.3389/fgene.2020.00101)
Supplement: Supplementary file 4 [file Table_3.docx]

**Supp. Table S3 -** Data quality summary.

| **Sample name** | **Raw reads** | **Clean reads** | **Raw bases** | **Clean bases** | **Error rate(%)** | **Q20(%)** | **Q30(%)** | **GC content (%)** |
| --- | --- | --- | --- | --- | --- | --- | --- | --- |
| II.3 | 64675368 | 62744214 | 9.7G | 9.4G | 0.02 | 96.52 | 91.41 | 60.11 |
| Control | 45600808 | 41455822 | 6.8G | 6.2G | 0.02 | 94.76 | 87.70 | 59.34 |

(1) Sample name: sample ID.

(2) Raw reads: reads count from the raw data, four rows as an unit, with statistics of reads count for every sequencing.

(3) Clean reads: Clean data is reads count filtered from raw data. Statistics method is similar with raw reads. All the following analysis is based on clean data.

(4) Raw bases: Base number of raw data. (number of raw reads) * (sequence length), converting unit to G.

(5) Clean bases: Base number of raw data after filtering. (number of clean reads) * (sequence length), converting unit to G.

(6) Error rate(%): base error rate of whole sequencing.

(7) Q20(%): Phred values greater than 20 base number contain the percentage of total bases. (Base number of Phred value > 20) / (Total base number)*100.

(8) Q30(%): Phred values greater than 30 base number contain the percentage of total bases. (Base number of Phred value > 30) / (Total base number)*100.

(9) GC content(%): The percentage of G&C base numbers of total bases.(G&C base number) / (Total base number)*100.
