# Supplementary material for: Skewed X-Chromosome Inactivation and Compensatory Upregulation of Escape Genes Precludes Major Clinical Symptoms in a Female With a Large Xq Deletion
Source: Front Genet. 2020 Mar 4;11:101. doi: 10.3389/fgene.2020.00101 (PMC7064548; doi:10.3389/fgene.2020.00101)
Supplement: Supplementary file 5 [file Table_4.docx]

| **Genomic Position on ChrX -hg19** | **Gene(s)** | **Region** | **ID** | **Reference allele** | **Alternative allele** | **HGVS*** | **Quality** | **Read Depth** | **II.3.AD** | **II.3.GT** | **Combined Status*** | **Sex bias**  **(Tukiaianen *et al*, 2017)** |
| --- | --- | --- | --- | --- | --- | --- | --- | --- | --- | --- | --- | --- |
| 1508324 | *SLC25A6/CRLF2* | PAR1 | . | G | A | NM_001636.3:c.408C>T | 2378.77 | 164 | 90,74 | G/A | escape | male-bias |
| 1508583 | *SLC25A6/CRLF2* | PAR1 | . | T | C | NM_001636.3:c.149A>G | 3317.77 | 193 | 93,1 | T/C | escape | male-bias |
| 30748284 | *GK* | Non PAR | rs5926945 | T | C | [NM_203391.3:c.*1425T>C](https://variantvalidator.org/variantvalidation/?variant=chrx%3A30748284T%3EC&primary_assembly=GRCh37&alignment=splign#variantValidator) | 382.77 | 11 | 0,11 | C/C | inactive | no significant bias |
| 41208310 | *DDX3X* | Non PAR | rs5963957 | A | C | [NM_001363819.1:c.*1338A>C](https://variantvalidator.org/variantvalidation/?variant=chrX%3A41208310A%3EC&primary_assembly=GRCh37&alignment=splign#variantValidator) | 1475.77 | 42 | 2,4 | C/C | escape | female-bias |
| 48418126 | *TBC1D25* | Non PAR | rs2293948 | A | G | [NM_001348262.1:c.878A>G](https://variantvalidator.org/variantvalidation/?variant=chrx%3A48418126A%3Eg&primary_assembly=GRCh37&alignment=splign#variantValidator) | 472.77 | 13 | 0,13 | G/G | inactive | male-bias |
| 48436507 | *RBM3* | Non PAR | rs8931 | C | G | [NM_006743.4:c.*678C>G](https://variantvalidator.org/variantvalidation/?variant=NM_006743.4:c.%2a678C%3eG&primary_assembly=GRCh37&alignment=splign#variantValidator) | 566.77 | 17 | 0,17 | G/G | variable | no significant bias |
| 48460314 | *WDR13* | Non PAR | rs235842 | A | G | [NR_029427.2:n.1459G=](https://variantvalidator.org/variantvalidation/?variant=NR_029427.2:n.1459G=&primary_assembly=GRCh37&alignment=splign#variantValidator) | 1244.77 | 34 | 0,33 | G/G | inactive | male-bias |
| 48690416 | *PCSK1N* | Non PAR | rs6520383 | G | T | [NM_013271.3:c.450C>A](https://variantvalidator.org/variantvalidation/?variant=chrX%3A48690416G%3ET&primary_assembly=GRCh37&alignment=splign#variantValidator) | 425.77 | 11 | 0,11 | T/T | unknown | no significant bias |
| 48751471 | *TIMM17B* | Non PAR | rs1128363 | A | G | [NM_001167947.1:c.378T>C](https://variantvalidator.org/variantvalidation/?variant=chrX%3A48751471A%3EG&primary_assembly=GRCh37&alignment=splign#variantValidator) | 1274.77 | 30 | 0,3 | G/G | inactive | no significant bias |
| 70784486 | *OGT/BCYRN1* | Non PAR | . | C | T | [NM_181673.2:c.2442C>T](https://variantvalidator.org/variantvalidation/?variant=chrX%3A70784486C%3ET&primary_assembly=GRCh37&alignment=splign#variantValidator) | 25.78 | 11 | 9,2 | C/T | inactive | female-bias |
| 71493691 | *RPS4X* | Non PAR | rs7580 | C | T | NM_001007.4:c.492G>A | 14481.77 | 581 | 179,402 | C/T | escape | female-bias |
| 73417059 | *FTX* | Non PAR | . | T | C | [NR_028379.1:n.695+77118A>G](https://variantvalidator.org/variantvalidation/?variant=NR_028379.1:n.695%2b77118A%3eG&primary_assembly=GRCh37&alignment=splign#variantValidator) | 28.769.999.999.999.900 | 11 | 9,2 | T/C | inactive | no significant bias |
| 73417096 | *FTX* | Non PAR | rs1935048 | C | T | [NR_028379.1:n.695+77081G>A](https://variantvalidator.org/variantvalidation/?variant=chrX%3A73417096c%3Et&primary_assembly=GRCh37&alignment=splign#variantValidator) | 385.77 | 12 | 0,11 | T/T | inactive | no significant bias |
| 101138792 | *ZMAT1* | Non PAR | rs5944882 | T | C | [NM_001282400.1:c.1094G=](https://variantvalidator.org/variantvalidation/?variant=NM_001282400.1:c.1094G=&primary_assembly=GRCh37&alignment=splign#variantValidator) | 505.77 | 15 | 0,15 | C/C | variable | female-bias |
| 117819773 | *DOCK11* | Non PAR | rs2379118 | A | C | [NM_144658.3:c.*3A>C](https://variantvalidator.org/variantvalidation/?variant=NM_144658.3:c.%2a3A%3eC&primary_assembly=GRCh37&alignment=splign#variantValidator) | 1278.77 | 31 | 0,31 | C/C | inactive | female-bias |
| 117819902 | *DOCK11* | Non PAR | rs2186106 | A | G | NM_144658.3:c.*132A>G | 889.77 | 24 | 0,24 | G/G | inactive | female-bias |
| 118603844 | *SLC25A5-AS1* | Non PAR | rs371749 | T | G | NM_001152.4:c.332T>G | 661.77 | 19 | 0,19 | G/G | inactive | heterogeneous |
| 118604436 | *SLC25A5-AS1* | Non PAR | rs12390 | T | C | NM_001152.4:c.699T>C | 1246.77 | 34 | 0,34 | C/C | inactive | heterogeneous |
| 128927890 | *SASH3* | Non PAR | rs859577 | T | C | NM_018990.3:c.*82T>C | 1030.77 | 28 | 0,28 | C | inactive | no significant bias |
| 148561332 | *IDS* | Non PAR | rs8965 | C | T | [NM_000202.7:c.*2945A=](https://variantvalidator.org/variantvalidation/?variant=NM_000202.7%3Ac.*2945A%3D&primary_assembly=GRCh37&alignment=splign#variantValidator) | 344.77 | 11 | 0,11 | T | inactive /unknown | no significant bias |
| 153278829 | *IRAK1* | Non PAR | rs1059703 | G | A | [NM_001025243.1:c.1358C>T](https://variantvalidator.org/variantvalidation/?variant=NM_001025243.1:c.1358C%3eT&primary_assembly=GRCh37&alignment=splign#variantValidator) | 511.77 | 12 | 0,12 | A | inactive | no significant bias |
| 154456747 | *VPB1* | Non PAR | rs572013 | A | G | [NM_003372.6:c.367G=](https://variantvalidator.org/variantvalidation/?variant=NM_003372.6:c.367G=&primary_assembly=GRCh37&alignment=splign#variantValidator) | 562.77 | 15 | 0,15 | G | inactive | male-bias |

**Supp. Table S4 –** Blood-expressed Single Nucleotide Polymorphisms (SNPs) on chromossome X from individual II.3.

*Nomenclature according to Human Genome Variation Society (HGVS). Reported XCI status refers to the XCI status in the combined list available in Tukiainen *et al.* (2017) from the studies of Carrel and Willard (2005) and Cotton *et al.* (2013); Genes within the Xq deletion are highlighted in yellow. AD: Allele Depth, the number of reads supporting either the reference genotype or SNP genotype; GT: genotype, the genotype of the locus.
