# Supplementary material for: Skewed X-Chromosome Inactivation and Compensatory Upregulation of Escape Genes Precludes Major Clinical Symptoms in a Female With a Large Xq Deletion
Source: Front Genet. 2020 Mar 4;11:101. doi: 10.3389/fgene.2020.00101 (PMC7064548; doi:10.3389/fgene.2020.00101)
Supplement: Supplementary file 6 [file Table_5.docx]

**Supp. Table S5 –** Blood-expressed indels on chromossome X from individual II.3.

| **Genomic Position on ChrX (hg19)** | **Gene** | **Region** | **ID** | **Reference allele** | **Quality** | **Read Depth** | **II.3.AD** | **II.3.GT** | **Combined Status*** | **Sex bias**  **(Tukiaianen et al, 2017)** |
| --- | --- | --- | --- | --- | --- | --- | --- | --- | --- | --- |
| 2659047 | *CD99* | PAR | G | GA | 588.73 | 55 | 31,21 | G/GA | escape | male-bias |
| 2659159 | *CD99* | PAR | A | AC | 445.73 | 36 | 12,19 | A/AC | escape | male-bias |
| 12995267 | *TMSB4X* | Non PAR | A | AT | 754.73 | 208 | 134,56 | A/AT | unknown | male-bias |
| 15844428 | *AP1S2* | Non PAR | C | CT | 120.73 | 32 | 22,8 | C/CT | escape | heterogeneous |
| 37672403 | *CYBB* | Non PAR | C | CT | 89.73 | 31 | 23,7 | C/CT | inactive | no significant bias |
| 41209153 | *DDX3X* | Non PAR | G | GT | 118,73 | 59 | 30,15 | G/GT | escape | female-bias |
| 48436466 | *RBM3* | Non PAR | C | CA | 219.73 | 28 | 13,13 | C/CA | variable | no significant bias |
| 48544198 | *WAS* | Non PAR | C | CA | 273.73 | 42 | 27,14 | C/CA | variable | no significant bias |
| 118377615 | *PGRMC1* | Non PAR | C | CT | 67.73 | 14 | 8,5 | C/CT | inactive | female-bias |
| 119739976 | *MCTS1* | Non PAR | C | CT | 49.73 | 14 | 8,4 | C/CT | inactive | no significant bias |
| 122755070 | *THOC2* | Non PAR | G | GT | 22.75 | 13 | 8,3 | G/GT | inactive | heterogeneous |

*Combined XCI status refers to the XCI status in the combined list from Carrel & Willard (2005) and Cotton et al (2013) reported by Tukiaianen et al (2017).
