# Supplementary material for: Skewed X-Chromosome Inactivation and Compensatory Upregulation of Escape Genes Precludes Major Clinical Symptoms in a Female With a Large Xq Deletion
Source: Front Genet. 2020 Mar 4;11:101. doi: 10.3389/fgene.2020.00101 (PMC7064548; doi:10.3389/fgene.2020.00101)
Supplement: Supplementary file 8 [file Table_7.docx]

**Supp. Table S7 -** Significant differential expressed X-linked genes between individual II.3 and control (hg19).

| **Gene ID** | **Gene Name** | **Strand** | **Start** | **End** | **Gene Length** | **II.3**  **read count** | **Control**  **read count** | **log2FoldChange**  **(II.3vsControl)** | **p adj**  **(II.3 vs Control)** | **Gene Type** | **Gene Description** |
| --- | --- | --- | --- | --- | --- | --- | --- | --- | --- | --- | --- |
| ENSG00000177324 | *BEND2* | - | 18181051 | 18239024 | 4611 | 1 | 60 | -5,3674 | 9,90E-02 | Protein coding | BEN_domain_containing_2 |
| ENSG00000198947 | *DMD* | - | 31137336 | 33357558 | 13956 | 3 | 41 | -3,3316 | 0.0030563 | Protein coding | dystrophin |
| ENSG00000216866 | *RPS2P55* | - | 40794198 | 40795117 | 883 | 68 | 15 | 2,5642 | 0.00063551 | Pseudogene | ribosomal_protein_S2_pseudogene_55 |
| ENSG00000270069 | *RP6-99M1.2* | - | 45604639 | 45629677 | 1758 | 28 | 4 | 3,1575 | 0.0038806 | lincRNA | - |
| ENSG00000017483 | *SLC38A5* | - | 48316920 | 48328644 | 2662 | 1250 | 400 | 2,0384 | 0.00029985 | Protein coding | solute_carrier_family_38_member_5 |
| ENSG00000102145 | *GATA1* | + | 48644962 | 48652718 | 1497 | 521 | 190 | 1,8494 | 0.0017455 | Protein coding | GATA_binding_protein_1_(globin_transcription_factor_1) |
| ENSG00000155659 | *VSIG4* | - | 65241580 | 65259967 | 2190 | 38 | 7 | 2,8106 | 0.0033926 | Protein coding | V-set_and_immunoglobulin_domain_containing_4 |
| ENSG00000147130 | *ZMYM3* | - | 70459474 | 70474996 | 6067 | 5 | 59 | -3,1384 | 0.00069215 | Protein coding | zinc_finger_MYM-type_3 |
| ENSG00000147138 | *GPR174* | + | 78426469 | 78427726 | 1258 | 0 | 27 | -7,5785 | 0.00053962 | Protein coding | G_protein-coupled_receptor_174 |
| ENSG00000124429 | *POF1B* | - | 84532402 | 84634748 | 3941 | 0 | 22 | -7,2847 | 0.0018453 | Protein coding | premature_ovarian_failure_1B |
| ENSG00000102290 | *PCDH11X* | + | 91034260 | 91878229 | 9176 | 0 | 23 | -7,3485 | 0.001308 | Protein coding | protocadherin_11_X-linked |
| ENSG00000123572 | *NRK* | + | 105066536 | 105202602 | 8064 | 0 | 20 | -7,1482 | 0.003638 | Protein coding | Nik_related_kinase |
| ENSG00000188153 | *COL4A5* | + | 107683074 | 107940775 | 6483 | 0 | 21 | -7,2181 | 0.0025819 | Protein coding | collagen_type_IV_alpha_5 |
| ENSG00000198918 | *RPL39* | - | 118920467 | 118925606 | 1915 | 5475 | 2166 | 1,7327 | 0.0024781 | Protein coding | ribosomal_protein_L39 |
| ENSG00000156920 | *GPR112* | + | 135383122 | 135519215 | 9931 | 0 | 30 | -7,7297 | 0.00020997 | Protein coding | G_protein-coupled_receptor_112 |
| ENSG00000130821 | *SLC6A8* | + | 152953554 | 152962048 | 3763 | 1116 | 397 | 1,8857 | 0.0010109 | Protein coding | solute_carrier_family_6_(neurotransmitter_transporter_creatine)_member_8 |
| ENSG00000165775 | *FUNDC2* | + | 154254255 | 154288578 | 6455 | 499 | 187 | 1,8101 | 0.0022556 | Protein coding | FUN14_domain_containing_2 |

Genes within the Xq25-q28 deletion are highlighted in yellow.
