# Supplementary material for: Skewed X-Chromosome Inactivation and Compensatory Upregulation of Escape Genes Precludes Major Clinical Symptoms in a Female With a Large Xq Deletion
Source: Front Genet. 2020 Mar 4;11:101. doi: 10.3389/fgene.2020.00101 (PMC7064548; doi:10.3389/fgene.2020.00101)
Supplement: Supplementary file 9 [file Table_8.docx]

**Supp. Table S8 -** Significant differential alternative splicing from X chromosome data (hg19). Note one significant alternative 5' splice site (A5SS) involving *HSD17B10* gene and two events of skipped exon on *XIST* and *IDS* genes alternative splicing.

| **Gene ID** | **Gene Symbol** | **Strand** | **Alternative Splicing event** | **Exon Start base** | **Exon end** | **Upstream ES** | **Upstream EE** | **Downstream ES** | **Downstream EE** | **IC SAMPLE 1** | **SC SAMPLE 1** | **IC SAMPLE 2** | **SC SAMPLE 2** | **Inc FormLen** | **Skip Form Len** | **p Value** | **FDR** |
| --- | --- | --- | --- | --- | --- | --- | --- | --- | --- | --- | --- | --- | --- | --- | --- | --- | --- |
| ENSG00000072506 | *HSD17B10* | - | 5' splice site  (A5SS) | 53458745 | 53458854 | 53458772 | 53458854 | 53458205 | 53458542 | 47 | 12 | 86 | 0 | 102 | 75 | 0.00021 | 0.0095 |
| ENSG00000229807 | *XIST* | - | skipped exon | 73041905 | 73042051 | 73040490 | 73040864 | 73045949 | 73046179 | 8 | 14 | 22 | 0 | 270 | 135 | 7.45778143907e-06 | 0.00106 |
| ENSG00000010404 | *IDS* | - | skipped exon | 148583604 | 148583707 | 148582479 | 148582568 | 148584841 | 148585019 | 0 | 21 | 18 | 19 | 139 | 82 | 0.00014 | 0.0074 |

Exon Start base: start position of skipped exon (SE) event; Exon End: end position of SE event; upstream exon skipped (ES): the start position of upstream exon in SE event; upstream EE: the end position of upstream exon in SE event; downstream ES: the start position of downstream exon in SE event; downstream EE: the end position of downstream exon in SE event; IJC_SAMPLE_1: inclusion junction counts for SAMPLE_1, with only the reads span splicing junctions taken into account; SJC_SAMPLE_1: exclusion junction counts for SAMPLE_1, with only the reads span splicing junctions taken into account; IJC_SAMPLE_2: inclusion junction counts for SAMPLE_2; SJC_SAMPLE_2: exclusion junction counts for SAMPLE_2; Inc Form Len: length of inclusion form, used for normalization; Skip Form Len: length of skipping form, used for normalization; FDR: the adjusted p-value
