# Supplementary material for: Skewed X-Chromosome Inactivation and Compensatory Upregulation of Escape Genes Precludes Major Clinical Symptoms in a Female With a Large Xq Deletion
Source: Front Genet. 2020 Mar 4;11:101. doi: 10.3389/fgene.2020.00101 (PMC7064548; doi:10.3389/fgene.2020.00101)
Supplement: Supplementary file 10 [file Table_9.docx]

**Supp. Table S9** - Comparative DE analysis amongst individual II.3 and the control groups (males and females), using the 117 blood-expressed genes within the deletion. Genes that exhibited significant adjusted p-values and |log2(FoldChange)| in at least one of the two comparisons are in bold.

| **Gene Name** | **Reported XCI status** | **II.3 x Males** | | | **II.3 x Females** | | |
| --- | --- | --- | --- | --- | --- | --- | --- |
|  |  | **log2(FoldChange)** | **p value** | **adjusted p value** | **log2(FoldChange)** | **p value** | **adjusted p value** |
| *ABCD1* | inactive | 0,065 | 0,869 | 0,936 | 0,771 | 0,276 | 0,492 |
| *AIFM1* | inactive | -0,86 | 0,205 | 0,39 | -1,079 | 0,104 | 0,28 |
| *ARHGAP4* | variable | -0,123 | 0,97 | 0,997 | 1,275 | 0,164 | 0,36 |
| *ARHGEF6* | inactive | -1,297 | 0,19 | 0,371 | -1,674 | 0,087 | 0,258 |
| *ATP11C* | inactive | 0,957 | 0,088 | 0,229 | 0,545 | 0,287 | 0,503 |
| *ATP6AP1* | inactive | 0,875 | 0,357 | 0,555 | 1,683 | 0,059 | 0,21 |
| *BCAP31* | inactive | 1,233 | 0,037 | 0,133 | 0,847 | 0,121 | 0,305 |
| *BCORL1* | inactive | -2,576 | 0,001 | 0,017 | -2,34 | 0,003 | 0,049 |
| *BRCC3* | inactive | -9,641 | 0 | 0,008 | -9,632 | 0 | 0,009 |
| *CD40LG* | variable | -0,72 | 0,55 | 0,724 | -1,492 | 0,184 | 0,386 |
| *CD99L2* | inactive | -0,237 | 0,891 | 0,948 | 0,272 | 0,653 | 0,806 |
| *CETN2* | inactive | 1,126 | 0,165 | 0,339 | 0,349 | 0,59 | 0,761 |
| *CXorf40A* | inactive | 1,708 | 0,04 | 0,14 | 1,914 | 0,011 | 0,099 |
| *CXorf40B* | inactive | 0,865 | 0,22 | 0,409 | 1,836 | 0,006 | 0,076 |
| *DDX26B* | inactive | 0,897 | 0,144 | 0,311 | 0,7 | 0,211 | 0,419 |
| *DKC1* | inactive | -1,937 | 0,057 | 0,175 | -1,987 | 0,048 | 0,191 |
| *DNASE1L1* | inactive | 0,595 | 0,559 | 0,73 | 1,019 | 0,341 | 0,558 |
| *ELF4* | inactive | -0,711 | 0,295 | 0,493 | -0,616 | 0,354 | 0,569 |
| *EMD* | unknown | 1,382 | 0,028 | 0,113 | 1,569 | 0,007 | 0,078 |
| *ENOX2* | inactive | -2,603 | 0,003 | 0,031 | -2,74 | 0,002 | 0,039 |
| *FAM122B* | variable | -0,506 | 0,431 | 0,625 | -0,534 | 0,394 | 0,605 |
| *FAM122C* | inactive | 0,156 | 0,776 | 0,878 | 0,926 | 0,171 | 0,368 |
| *FAM127A* | inactive | 0,48 | 0,435 | 0,629 | 1,197 | 0,043 | 0,182 |
| *FAM127B* | inactive | -0,353 | 0,696 | 0,826 | 0,288 | 0,616 | 0,778 |
| *FAM3A* | inactive | 1,453 | 0,153 | 0,323 | 2,299 | 0,013 | 0,103 |
| *FAM50A* | inactive | 0,568 | 0,24 | 0,431 | 0,615 | 0,172 | 0,37 |
| *FAM58A* | inactive | 1,489 | 0,138 | 0,303 | 1,622 | 0,08 | 0,247 |
| *FHL1* | inactive | 0,026 | 0,918 | 0,964 | -0,209 | 0,841 | 0,917 |
| *FLNA* | inactive | -2,298 | 0,052 | 0,164 | -0,229 | 0,914 | 0,961 |
| *FMR1* | inactive | -1,69 | 0,102 | 0,25 | -2,206 | 0,033 | 0,162 |
| ***FUNDC2*** | **inactive** | **3,468** | **0** | **0** | **2,64** | **0** | **0** |
| *G6PD* | inactive | 1,449 | 0,125 | 0,286 | 1,765 | 0,039 | 0,174 |
| *GAB3* | inactive | -3,551 | 0,001 | 0,019 | -2,956 | 0,006 | 0,074 |
| *GDI1* | inactive | -0,948 | 0,119 | 0,276 | -0,443 | 0,464 | 0,665 |
| *HCFC1* | variable | -3,941 | 0,003 | 0,033 | -1,074 | 0,393 | 0,604 |
| *HMGB3* | inactive | -7,746 | 0,03 | 0,118 | -7,47 | 0,046 | 0,188 |
| *HPRT1* | inactive | 1,16 | 0,055 | 0,172 | 0,719 | 0,194 | 0,397 |
| *HTATSF1* | inactive | -0,033 | 1 | 1 | -0,563 | 0,563 | 0,743 |
| *IDH3G* | inactive | 0,616 | 0,33 | 0,529 | 1,058 | 0,076 | 0,24 |
| *IDS* | inactive | -0,627 | 0,487 | 0,672 | -0,763 | 0,382 | 0,594 |
| *IKBKG* | escape | 0,009 | 0,909 | 0,96 | 0,644 | 0,403 | 0,612 |
| *IRAK1* | inactive | 0,336 | 0,651 | 0,797 | 1,74 | 0,029 | 0,152 |
| *LAGE3* | inactive | 2,123 | 0,216 | 0,403 | 2,793 | 0,07 | 0,23 |
| *MAP7D3* | inactive | -1,067 | 0,398 | 0,595 | -1,616 | 0,193 | 0,396 |
| *MBNL3* | inactive | 1,571 | 0,016 | 0,083 | 0,844 | 0,158 | 0,353 |
| ***MCF2*** | **inactive** | **3,822** | **0** | **0,003** | 2,273 | 0,011 | 0,096 |
| *MECP2* | inactive | 1,038 | 0,067 | 0,193 | 0,68 | 0,192 | 0,395 |
| *MMGT1* | inactive | -1,458 | 0,358 | 0,558 | -1,459 | 0,39 | 0,602 |
| *MOSPD1* | inactive | -0,892 | 0,437 | 0,63 | -1,654 | 0,105 | 0,282 |
| *MPP1* | inactive | 1,204 | 0,069 | 0,196 | 0,654 | 0,275 | 0,49 |
| *MST4* | inactive | 0,693 | 0,263 | 0,457 | 0,077 | 0,827 | 0,908 |
| *MTM1* | inactive | -0,794 | 0,396 | 0,593 | -1,36 | 0,129 | 0,314 |
| *MTMR1* | inactive | -3,757 | 0,001 | 0,013 | -3,535 | 0,001 | 0,032 |
| *NAA10* | variable | 1,315 | 0,048 | 0,156 | 0,957 | 0,127 | 0,312 |
| *NSDHL* | inactive | -3,909 | 0,002 | 0,022 | -3,971 | 0,001 | 0,035 |
| *OCRL* | inactive | -0,273 | 0,778 | 0,88 | 0,164 | 0,751 | 0,866 |
| *PDZD4* | inactive | -2,391 | 0,059 | 0,178 | -0,344 | 0,873 | 0,937 |
| *PHF6* | inactive | 0,663 | 0,236 | 0,426 | 0,135 | 0,749 | 0,865 |
| *PLXNA3* | inactive | -5,054 | 0,001 | 0,015 | -2,242 | 0,122 | 0,305 |
| *RAB33A* | inactive | 1,115 | 0,227 | 0,417 | 1,072 | 0,226 | 0,436 |
| *RAP2C* | inactive | 1,083 | 0,036 | 0,132 | 0,823 | 0,087 | 0,258 |
| *RBMX* | inactive | 0,115 | 0,802 | 0,896 | -0,277 | 0,734 | 0,855 |
| *RBMX2* | inactive | -0,179 | 0,876 | 0,94 | -0,876 | 0,272 | 0,487 |
| *RENBP* | escape | 1,142 | 0,251 | 0,443 | 1,255 | 0,167 | 0,364 |
| *RP13-507I23.1* | na | -1,053 | 0,506 | 0,687 | 0,269 | 0,735 | 0,856 |
| *RP3-527F8.2* | na | 2,588 | 0,001 | 0,013 | 1,786 | 0,01 | 0,092 |
| *RPL10* | inactive | 2,432 | 0,044 | 0,149 | 2,36 | 0,026 | 0,144 |
| *SASH3* | inactive | -0,287 | 0,696 | 0,826 | -0,24 | 0,75 | 0,865 |
| *SH2D1A* | variable | 1,132 | 0,064 | 0,188 | 0,637 | 0,252 | 0,466 |
| *SLC10A3* | inactive | 1,487 | 0,114 | 0,269 | 2,116 | 0,017 | 0,119 |
| *SLC25A14* | inactive | 0,308 | 0,622 | 0,778 | 0,001 | 0,932 | 0,973 |
| ***SLC6A8*** | **unknown** | **7,147** | **0** | **0,001** | 4,031 | 0,001 | 0,034 |
| *SLC9A6* | inactive | -9,897 | 0,001 | 0,017 | -9,088 | 0,003 | 0,049 |
| *SSR4* | inactive | 2,681 | 0,027 | 0,113 | 2,599 | 0,014 | 0,109 |
| *STAG2* | inactive | 1,324 | 0,101 | 0,249 | 0,41 | 0,536 | 0,723 |
| *TAZ* | inactive | -0,808 | 0,473 | 0,661 | 0,239 | 0,703 | 0,835 |
| *TENM1* | inactive | -1,18 | 0,197 | 0,381 | -0,471 | 0,648 | 0,802 |
| *TKTL1* | inactive | -0,77 | 0,611 | 0,769 | -0,022 | 0,904 | 0,956 |
| *TMEM185A* | inactive | -1,382 | 0,295 | 0,492 | -0,164 | 1 | 1 |
| *TMLHE* | inactive | -2,24 | 0,046 | 0,154 | -2,509 | 0,02 | 0,129 |
| *UBL4A* | inactive | 0,056 | 0,845 | 0,923 | 0,714 | 0,449 | 0,653 |
| *UTP14A* | inactive | -0,699 | 0,403 | 0,6 | -0,853 | 0,297 | 0,513 |
| ***VBP1*** | **inactive** | **2,894** | **0** | **0** | 1,983 | 0 | 0,014 |
| *VMA21* | inactive | -0,558 | 0,393 | 0,59 | -0,56 | 0,384 | 0,596 |
| *XIAP* | inactive | -0,267 | 0,719 | 0,842 | -0,336 | 0,64 | 0,798 |
| *XPNPEP2* | inactive | -8,673 | 0,01 | 0,063 | -8,029 | 0,014 | 0,11 |
| *ZDHHC9* | inactive | -8,954 | 0,001 | 0,013 | -8,789 | 0,001 | 0,03 |
| *ZNF185* | inactive | -1,24 | 0,072 | 0,201 | -0,629 | 0,36 | 0,575 |
| *ZNF280C* | variable | -1,56 | 0,155 | 0,326 | -1,882 | 0,067 | 0,224 |
| *ZNF75D* | inactive | -1,09 | 0,458 | 0,648 | -0,79 | 0,657 | 0,807 |

Genes that showed evidence of low or no expression (CPM < 1 in at least 50% of the samples) were removed from the table.
